# Surrogate endpoints for overall survival in randomized clinical trials testing immune checkpoint inhibitors: a systematic review and meta-analysis

**DOI:** 10.3389/fimmu.2024.1340979

**Published:** 2024-01-29

**Authors:** Isabella Sala, Eleonora Pagan, Laura Pala, Chiara Oriecuia, Marco Musca, Claudia Specchia, Tommaso De Pas, Javier Cortes, Giuseppe Giaccone, Michael Postow, Richard D. Gelber, Vincenzo Bagnardi, Fabio Conforti

**Affiliations:** ^1^ Department of Statistics and Quantitative Methods, University of Milan-Bicocca, Milan, Italy; ^2^ Department of Medicine and Surgery, University of Milan-Bicocca, Milan, Italy; ^3^ Department of Medical Oncology, Humanitas Gavazzeni, Bergamo, Italy; ^4^ Department of Clinical and Experimental Sciences, University of Brescia, Brescia, Italy; ^5^ Department of Molecular and Translational Medicine, University of Brescia, Brescia, Italy; ^6^ Methodology for Clinical Research Laboratory, Istituto di Ricerche Farmacologiche Mario Negri Istituto di Ricovero e Cura a Carattere Scientifico (IRCCS), Milan, Italy; ^7^ International Breast Cancer Center, Pangaea Oncology, Quiron Group, Madrid, Spain; ^8^ International Breast Cancer Center, Pangaea Oncology, Quiron Group, Barcelona, Spain; ^9^ Faculty of Biomedical and Health Sciences, Department of Medicine, Universidad Europea de Madrid, Madrid, Spain; ^10^ Meyer Cancer Center, Weill Cornel Medicine, New York, NY, United States; ^11^ Department of Medicine, Memorial Sloan Kettering Cancer Center and Weill Cornell Medical College, New York, NY, United States; ^12^ Department of Data Science, Dana-Farber Cancer Institute, Harvard Medical School, Harvard Tseng-Hsi (T.H.) Chan School of Public Health, and Frontier Science and Technology Research Foundation, Boston, MA, United States; ^13^ University of Milan, Milan, Italy

**Keywords:** immunotherapy, surrogate, randomized clinical trial, immune check inhibitor (ICI), methodology

## Abstract

**Introduction:**

There is debate on which are the best surrogate endpoint and metric to capture treatment effect on overall survival (OS) in RCTs testing immune-checkpoint inhibitors (ICIs).

**Methods:**

We systematically searched for RCTs testing ICIs in patients with advanced solid tumors. Inclusion criteria were: RCTs i) assessing PD-(L)1 and CTLA-4 inhibitors either as monotherapy or in combination with another ICI, and/or targeted therapy, and/or chemotherapy, in patients with advanced solid tumors; ii) randomizing at least 100 patients. We performed a meta-analysis of RCTs to compare the surrogacy value of PFS and modified-PFS (mPFS) for OS in RCTs testing ICIs, when the treatment effect is measured by the hazard ratio (HR) for OS, and by the HR and the ratio of restricted mean survival time (rRMST) for PFS and mPFS.

**Results:**

61 RCTs (67 treatment comparisons and 36,034 patients) were included in the analysis. In comparisons testing ICI plus chemotherapy, HR_PFS_ and HR_mPFS_ both had a strong surrogacy value (R^2^ = 0.74 and R^2^ = 0.81, respectively). In comparisons testing ICI as monotherapy, HR_PFS_ was the best surrogate, although having a moderate correlation (R^2^ = 0.58). In comparisons testing ICI plus other treatment(s), the associations were very weak for all the surrogate endpoints and treatment effect measures, with R^2^ ranging from 0.01 to 0.22.

**Conclusion:**

In RCTs testing ICIs, the value of potential surrogates for HR_OS_ was strongly affected by the type of treatment(s) tested. The evidence available supports HR_PFS_ as the best surrogate, and disproves the use of alternative endpoints, such as the mPFS, or treatment effect measures, such as the RMST.

## Introduction

Overall survival (OS) is the gold-standard endpoint used to demonstrate the clinical efficacy of new cancer drugs in randomized clinical trials (RCTs). The primary effect measure of interest is the ratio of the hazards of death, namely the OS hazard ratio (HR_OS_), assessed over the entire follow-up period (FUP) and estimated using the proportional hazards (PH) Cox model.

However, a reliable estimation of HR_OS_ requires large RCTs with long FUP, resulting in increase in costs and time required before a new cancer drug is available to patients. To expedite drug approvals, the evaluation of new treatments in RCTs often relies on the assessment of their effects on surrogate endpoints, under the assumption that these effects accurately predict those on OS at the final analysis (1).

Progression-free survival (PFS) has been long used as a surrogate endpoint for OS in RCTs testing chemotherapy and targeted therapy in patients with advanced solid tumors. Also, the HR estimated from a Cox PH model for PFS (HR_PFS_) is routinely used as a measure to empirically compare experimental versus control arms.

The weak correlation (R^2^<0.40) between HR_PFS_ and HR_OS_ resulting in RCTs testing immune checkpoint inhibitors (ICIs) (2, 3) may challenge the belief that HR_PFS_ is a potential surrogate endpoint for OS. However, the poor correlation between HR_PFS_ and HR_OS_ may be attributed to ICIs’ novel mechanisms for activating or rehabilitating self-immunity against tumors, which could result in delayed clinical effects and long-term responders, as well as in disease progression followed by tumor shrinkage (pseudo-progression). In these instances, the PFS curves may take some time to separate, and the immunotherapy agent curve may have a long tail, leading to the violation of the PH assumption on which the calculation of HR_PFS_ is based (4).

The restricted mean survival time (RMST), namely the mean survival time to some prespecified time point t^*^, was proposed as an alternative treatment effect measure to address both delayed response and long-term responders issues, accounting for deviation from PH assumption (5). The treatment effect on PFS can be measured as the ratio of RMST (rRMST), which is the ratio of the area under the Kaplan-Meier (KM) PFS curve for the control group *vs* experimental group, from time 0 to a chosen time t^*^.

Early overlapping PFS curves may also depend on pseudo-progression events, a documented type of response to ICIs that occur when an initial apparent RECIST-defined progression is observed prior to eventual disease improvement. To consider pseudo-progressions, Wang et al. (6) recently proposed a novel endpoint, the modified PFS (mPFS), which omits the events of disease progression (but not deaths) within *n* months (e.g., 3 months) after randomization, showing that HR_mPFS_ outperformed HR_PFS_ as surrogate for HR_OS_ in ICI trials.

Here, we performed a systematic review and meta-analysis of RCTs testing ICIs in patients with advanced solid tumors to compare the surrogacy value for HR_OS_ of both PFS and mPFS as endpoints and HR and rRMST as treatment effect measures, in strata of type of treatment administered in the experimental arm [i.e., ICI alone, ICI plus chemotherapy, ICI plus ICI or other treatment(s)].

## Methods

The value of PFS and mPFS as surrogate endpoint for OS in RCTs testing ICIs was assessed using a meta-analytical approach based on pseudo individual patient-level data (IPD) (see details below). The treatment effect was measured by the HR for OS, and by the HR and the rRMST for the two surrogate endpoints.

### Search strategy, selection criteria and data extraction

We followed recommendations of the Preferred Reporting Items for Systematic reviews and Meta-Analyses (PRISMA) (7) and the Reporting of Surrogate Endpoint Evaluation using Meta-analyses (ReSEEM) (8) guidelines to perform this systematic review and meta-analysis. We searched PubMed, Embase, and Scopus for phase II or III RCTs testing ICIs, published from the inception of each database to December 31, 2021. We also reviewed abstracts and presentations from all major conference proceedings, including the American Society of Clinical Oncology and the European Society for Medical Oncology, from January 2010 to December 2021.

Two investigators (LP and FC) independently searched the databases. The search terms were “CTLA-4”, “cytotoxic T-lymphocyte-associated protein 4”, “PD-1”, “programmed death receptor 1”, “PD-L1”, “immune checkpoint inhibitor”, “ipilimumab”, “tremelimumab”, “nivolumab”, “pembrolizumab”, “durvalumab”, “atezolizumab”, “cemiplimab”, “spartalizumab”, “avelumab”, “toripalimab”, “dostarlimab”, “balstilimab”, “penpulimab”, “retifanlimab”, “sintilimab”.

We included RCTs: i) assessing PD-1, PD-L1 and CTLA-4 inhibitors either as monotherapy or in combination with another ICI, and/or targeted therapy, and/or anti-angiogenesis drugs, and/or chemotherapy, in patients with advanced solid tumors; ii) randomizing at least 100 patients; iii) displaying the KM survival curves for OS and PFS.

We excluded single-arm phase I and II trials (i.e., non-randomized trials), RCTs conducted in (neo)adjuvant setting or in hematologic malignancies, and RCTs considering ICIs as control arm (either monotherapy or combined with other therapies).

Titles, abstracts, and full-text articles were reviewed independently by four authors (FC, LP, EP, IS). Inconsistencies were discussed by all authors to reach consensus. Reference lists of articles included in the final selection were reviewed to identify additional relevant papers. When duplicate publications were identified, only the most recent and complete were included.

Based on a predefined form, we extracted data on the following variables: study name, first author and year of publication, study design and blinding, trial phase, primary endpoint(s), underlying malignancy, number of patients, median FUP time, line of therapy, type of experimental and control treatment.

### Quality assessment of trials

To ascertain risk of bias, we assessed the methodological quality of each trial using the Cochrane Risk of bias tool (version 5.2.0) (9). Responses in each domain (random sequence generation, allocation concealment, blinding of participants and personnel, blinding of outcome assessment, incomplete outcome data and selective outcome reporting) were assessed as having a ‘low’, ‘unclear’ or ‘high’ risk of bias.

### Individual patient-level data reconstruction

Pseudo IPD for PFS and OS were reconstructed from the published KM curves. We used a web based validated tool [WebPlotDigitizer (10)] to extract data coordinates from published KM curves. Then, pseudo IPD were reconstructed using the validated algorithm proposed by Guyot et al. (11).

To derive mPFS, disjointed PFS and OS pseudo IPD were matched using a simulation-based algorithm, as described in Wang et al. (6). Briefly, the algorithm matches PFS-OS pseudo IPD under the following conditions: i) for a given patient, the PFS duration should not exceed the OS duration; ii) patients with events in the OS pseudo IPD dataset should be a subgroup of patients with events in the PFS pseudo IPD dataset. Given that these requirements are insufficient to accurately capture the original matched PFS-OS IPD, we simulated 1000 qualified datasets of matched PFS-OS pseudo IPD for each included treatment arm.

### Statistical analysis

The data extracted from the included three-arm trials were treated as two separate comparisons, with the control arm being duplicated in both comparisons. For this reason, our unit of analysis was the comparison between pairs of treatment arms, and not the trial. Each pairwise comparison was categorized according to the type of treatment administered in the experimental arm: ICI alone, ICI plus chemotherapy, or ICI plus ICI or other treatment(s).

For each comparison, we used the reconstructed pseudo IPD to estimate HR_OS_, HR_PFS_, HR_mPFS_, rRMST_PFS_, rRMST_mPFS_ with their 95% confidence intervals (95% CI). rRMST was obtained as the ratio of the RMST in the control group to the RMST in the experimental group. Both an rRMST and an HR less than one favored the experimental treatment.

Within each treatment comparison, we compared differences in treatment effect measures by using the ratio of the surrogate effect measure (*s*; i.e., HR_PFS_, HR_mPFS_, rRMST_PFS_, or rRMST_mPFS_) to the final effect measure (*f*; i.e., HR_OS_). A ratio $\frac{s}{f}<1$ indicated that the treatment effect size on the surrogate measure overestimates the treatment effect size on the final measure.

The pairwise agreement between the statistical significance of HR_OS_ and each surrogate measure was assessed. In our analysis a p-value below 0.05 was considered statistically significant, which would in turn indicate the potential for drug approval. Cohen’s kappa coefficient was used to measure the agreement between HR_OS_ and each surrogate. Additionally, McNemar’s statistic was used to test the null hypothesis that both measures provided a similar proportion of statistically significant findings.

Meta-analyses of the treatment effect measures were conducted using random effects models. A weighted linear regression (WLS) model was used to quantify the association between the treatment effect on the final endpoint (HR_OS_) and each surrogate measure (HR_PFS_, HR_mPFS_, rRMST_PFS_, and rRMST_mPFS_). The coefficient of determination (R^2^) was used to quantify the surrogacy value at trial-level of each potential surrogate endpoint. The 95% CI for R^2^ was estimated by bootstrap analysis with 1000 samples. According to ReSEEM guidelines (8), R^2^ values equal to or higher than 0.7 represent strong correlations (and was therefore suggestive of surrogacy), values between 0.69 and 0.5 represent moderate correlations, and values lower than 0.5 represent weak correlations. The slope of the regression line was also reported as an alternative measure of surrogacy. For the treatment effects to be associated, we required that the slope significantly differed from zero. Finally, we calculated the surrogate threshold effect (STE), defined as the minimum treatment effect on the surrogate endpoint necessary to predict a significant OS benefit in a future trial.

A comprehensive description of statistical analysis was included in the **Supplementary Material**.

Analyses were performed with SAS software v9.4 (SAS Institute, Cary, North Carolina, USA) and R software v3.6.0.

### Patient and public involvement

Members of the study group have regular meetings with patient representatives about ongoing scientific projects and activities. During these meetings the project and its objectives are discussed, and we accepted the patients’ suggestions, which were mainly focused on the need to make the final version of the paper as clear and less technical as possible, to widely disseminate the results given the relevant implications for research and clinical practice.

## Results

Overall, 61 RCTs comprising a total of 36,034 patients were included in the analysis (**Supplementary Figure S2**, **Supplementary Table S1**).

**Table 1** shows the main features of included trials (12–78). Six phase I/II or II or II/III trials (10%) and 55 phase III trials (90%) were included. The publication years spanned from 2011 to 2021, with 29 (48%) published in 2021. Forty-one studies (67%) were in the first-line setting and 27 (44%) enrolled patients with non-small cell lung cancer. OS alone was the primary endpoint in 25 (41%) trials, PFS alone in 12 trials (20%), OS and PFS or OS and overall response rate (ORR) as co-primary in 22 (36%) and 1 trial (2%), respectively. The sole phase I/II included trial used ORR alone as primary endpoint.

**Table 1:** Main features of included trials.

*N of included trials*	N (%)
	*61*
Year of publication	
2011-2020	32 (52.5)
2021	29 (47.5)
Phase	
I/II or II or II/III	6 (9.8)
III	55 (90.2)
Line of therapy	
I	41 (67.2)
>I	20 (32.8)
Primary endpoint	
OS	25 (41.0)
PFS	12 (19.7)
OS+PFS	22 (36.1)
OS+ORR	1 (1.6)
ORR	1 (1.6)
Median follow-up time (range)	18.9 (5.1-69.5)
Tumor site	
Breast cancer	3 (4.9)
Colorectal cancer	1 (1.6)
Esophageal cancer	3 (4.9)
Gastric cancer	3 (4.9)
Head and neck cancer	4 (6.6)
Melanoma	5 (8.2)
Mesothelioma	2 (3.3)
Non-small cell lung cancer	27 (44.3)
Renal cancer	5 (8.2)
Small cell lung cancer	4 (6.6)
Urothelial cancer	4 (6.6)

OS, overall survival; PFS, progression-free survival; ORR, overall response rate.

The median FUP of trials was 18.9 months, ranging from 5.1 to 69.5 months.

Six trials had three treatment arms, for a total of 67 comparisons analyzed (**Table 2**). Thirty comparisons (45%) tested ICI alone, 22 comparisons (33%) evaluated the combination of ICI with chemotherapy, and 15 comparisons (22%) tested other ICI(s)-containing combinations, including an anti-PD-(L)1 combined with anti-angiogenesis drugs in 5 trials, the combination of an anti-PD-(L)1 with an anti-CTLA-4 drug in 7 trials, combo-immunotherapy (i.e., anti-PD-(L)1 plus anti-CTLA-4) plus chemotherapy in 2 trials, and an anti-PD-L1 combined with targeted therapy in one trial. The treatment administered in the control arm was chemotherapy alone in 56 comparisons (84%), anti-angiogenesis agent alone or in combination with chemotherapy in 6 comparisons (9%), targeted therapy in 2 comparisons (3%) and placebo in 3 comparisons (5%). Chemotherapy was administered as control arm in all the comparisons testing ICI plus chemotherapy, in 27 (90%) comparisons testing ICI alone, and in 7 (47%) comparisons testing ICI plus ICI or other treatment(s).

**Table 2:** Main features of included comparisons.

*N of included comparisons*	Type of treatment administered in the experimental arm			Total
	ICI alone	ICI + CT	ICI + ICI or other treatment(s)	
	N (%)	N (%)	N (%)	N (%)
	*30*	*22*	*15*	*67*
Type of treatment administered in the control arm				
CT	27 (90.0)	22 (100.0)	7 (46.7)	56 (83.6)
Anti-angiogenesis agent	0	0	4 (26.7)	4 (6.0)
Anti-angiogenesis agent + CT	0	0	2 (13.3)	2 (3.0)
Targeted therapy	1 (3.3)	0	1 (6.7)	2 (3.0)
Placebo	2 (6.7)	0	1 (6.7)	3 (4.5)
PH assumption was not rejected for ^a^				
Both OS and PFS	5 (16.7)	18 (81.8)	8 (53.3)	31 (46.3)
OS or PFS only	13 (43.3)	3 (13.6)	1 (6.7)	17 (25.4)
None	12 (40.0)	1 (4.5)	6 (40.0)	19 (28.4)

ICI, immune checkpoint inhibitor; CT, chemotherapy; OS, overall survival; PFS, progression-free survival; PH, proportional hazards.

a The Grambsch-Therneau test was used to test the PH assumption. PH assumption was not rejected (p-value greater than 0.05):

- for both OS and PFS: ICI alone, n=5; ICI+CT, n=18; ICI+ICI or other treatment(s), n=8.

- for OS only: ICI alone, n=13; ICI+CT, n=1; ICI+ICI or other treatment(s), n=1.

- for PFS only: ICI alone, n=0; ICI+CT, n=2; ICI+ICI or other treatment(s), n=0.

- for none: ICI alone, n=12; ICI+CT, n=1; ICI+ICI or other treatment(s), n=6.

The proportional hazards assumption was not rejected (i.e. Grambsch-Therneau test p-value greater than 0.05) for both OS and PFS in 31 out of 67 comparisons (46%), for OS only in 15 comparisons (22%), for PFS only in 2 comparisons (3%), and neither for OS nor PFS in 19 comparisons (28%).

The PH assumption held for both OS and PFS in 82% of comparisons testing ICI plus chemotherapy, in 53% of comparisons testing ICI plus ICI or other treatment(s), and in only 5 (17%) comparisons testing ICI alone.

**Supplementary Table S2** reports the quality assessment of trials according to the Cochrane Risk of bias tool. Overall, the quality of trials was high, as the risks of selection, attrition, reporting and other forms of bias for all the RCTs included in the analysis were low. The only potential biases affecting trials were performance and detection bias, since only 21 out of 61 RCTs had a double blinding design.

**Figure 1:**
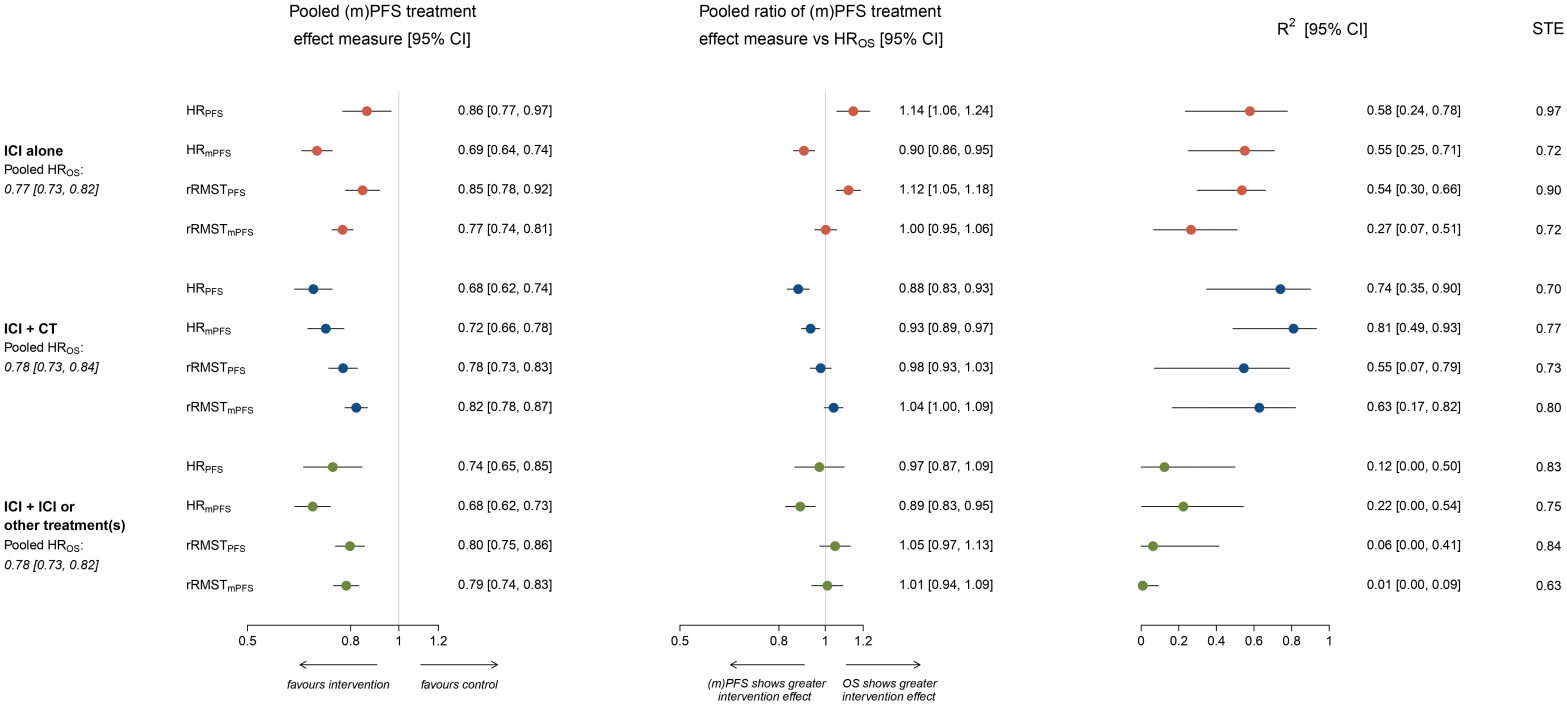
Forest plots showing meta-analytic pooled estimate (with 95% CI) of treatment effects on OS and potential surrogate endpoints, meta-analytic pooled estimate (with 95% CI) of the ratio between surrogate endpoint and HR_OS_, R^2^ coefficient (with 95% CI) from the weighted linear regression and surrogate threshold effect (STE), by type of treatment administered in the experimental arm. The figure shows in the left panel the meta-analytic pooled estimate (circles) of treatment effects on potential surrogate endpoints, by type of treatment administered in the experimental arm. Horizontal lines indicate the 95% CI and the solid vertical line indicates a HR or rRMST of 1, which is the null-hypothesis value. Values <1 indicate a treatment effect in favor of the experimental arm, while values >1 indicate treatment effects in favor of the control. The meta-analytic pooled estimates of HR_OS_ are also displayed. The central panel shows the meta-analytic pooled estimate (circles) of the ratio between surrogate endpoint and HR_OS_, by type of treatment administered in the experimental arm. Horizontal lines indicate the 95% CI, and the solid vertical line indicates a ratio of 1, which is the null-hypothesis value. Values <1 indicate a surrogate endpoint that overestimates the protective treatment effect size observed with HR_OS_, while values >1 indicate a surrogate endpoint that underestimates it. The right panel shows the R^2^ coefficient (with 95% CI) estimated from the weighted linear regression model, by type of treatment administered in the experimental arm. Surrogate threshold effect (STE) values are also reported on the right.

**Supplementary Table S3** reports the treatment effect estimates and their ratios derived from pseudo IPD extracted from the published KM curves for each included comparison. The HRs for OS ranged between 0.43 and 1.03 in comparisons testing ICI alone, between 0.56 and 1.26 in comparisons testing ICI plus CT, and between 0.59 and 0.86 in comparisons testing ICI plus ICI or other treatment(s). Notably, pooled HR_OS_ were very similar across strata: HR_OS_=0.77 (95% CI: 0.73, 0.82) for ICI alone, HR_OS_=0.78 (95% CI: 0.73, 0.84) for ICI plus chemotherapy, and HR_OS_=0.78 (95% CI: 0.73, 0.82) for ICI plus ICI or other treatment(s) (**Figure 1**).

Pooled HRs for PFS and mPFS varied according to the type of treatment administered in the experimental arm. Pooled HR_PFS_=0.86 (95% CI: 0.77, 0.97) in ICI alone, HR_PFS_=0.68 (95% CI: 0.62, 0.74) in ICI plus chemotherapy, and HR_PFS_=0.74 (95% CI: 0.65, 0.85) in ICI plus ICI or other treatment(s). Pooled HR_mPFS_ was 0.69 (95% CI: 0.64, 0.74), 0.72 (95% CI: 0.66, 0.78) and 0.68 (95% CI: 0.62, 0.73) respectively (**Figure 1**).

Across the 67 comparisons, the time horizons t^*^ defining RMST ranged from 8.7 months to 5.1 years (median 20.9 months [IQR: 15.0, 26.0]) and no important differences emerged between strata.

Pooled rRMST_PFS_ and rRMST_mPFS_ were similar within and across strata of treatment administered in the experimental arm. In comparisons testing ICI alone pooled rRMST_PFS_=0.85 (95% CI: 0.78, 0.92) and rRMST_mPFS_=0.77 (95% CI: 0.74, 0.81), in comparisons testing ICI plus chemotherapy pooled rRMST_PFS_=0.78 (95% CI: 0.73, 0.83) and rRMST_mPFS_=0.82 (95% CI: 0.78, 0.87), while in comparisons testing ICI plus ICI or other treatment(s) pooled rRMST_PFS_=0.80 (95% CI: 0.75, 0.86) and rRMST_mPFS_=0.79 (95% CI: 0.74, 0.83; **Figure 1**).

When all the RCTs were pooled together, none of the endpoints (i.e., PFS and mPFS) or metrics (i.e., HR and RMST) investigated had a strong association with HR_OS_: the R^2^ of the association with HR_OS_ was respectively 0.38 (95% CI: 0.23, 0.52) for HR_PFS_, 0.60 (95% CI: 0.39-0.73) for HRm_PFS_, 0.39 (95% CI: 0.23, 0.52) for rRMST_PFS,_ and 0.31 (95% CI: 0.11, 0.50) for rRMST_mPFS._

**Figure 1** shows the pooled ratio between each surrogate treatment effect measure and HR_OS_ by type of treatment administered in the experimental arm.

Notably, The HR_PFS_ showed a larger treatment effect for the experimental treatment compared to that observed for HR_OS_ (ratio of the two treatment effect measures <1) in 7 (23%) comparisons testing ICI alone, in 20 (91%) comparisons testing ICI plus chemotherapy, and in 8 (53%) comparisons testing ICI plus ICI or other treatment(s) (**Supplementary Table S3**).

Overall, the HR_PFS_ significantly underestimated the protective treatment effect size observed on HR_OS_ in comparisons testing ICI alone (pooled ratio HR_PFS_/HR_OS_=1.14, 95% CI: 1.06, 1.24) and, on the contrary, significantly overestimated it in comparisons testing the combination of ICI plus chemotherapy (pooled ratio HR_PFS_/HR_OS_=0.88, 95% CI: 0.83, 0.93).

Conversely, the HR_mPFS_ significantly overestimated the protective treatment effect size observed on HR_OS_ regardless of the type of treatment administered in the experimental arm (pooled ratio HR_mPFS_/HR_OS_ was 0.90 (95% CI: 0.86, 0.95) for ICI alone, 0.93 (95% CI: 0.89, 0.97) for ICI plus chemotherapy, and 0.89 (95% CI: 0.83-0.95) for ICI plus ICI or other treatment(s); **Figure 1**).

**Supplementary Table S4** shows the pairwise agreement between the statistical significance of HR_OS_ and each surrogate measure. In comparisons testing ICI alone, there was no agreement between statistical significance of HR_OS_ and HR_PFS_ (Cohen’s Kappa coefficient was 0.02 [95% CI: -0.32, 0.36] and McNemar’s test p-value 0.197), while a stronger agreement was observed between HR_OS_ and the other surrogate measures (Cohen’s Kappa coefficient ranged from 0.30 for HR_OS_ vs rRMST_PFS_ [McNemar’s test p-value = 0.035] to 0.51 for HR_OS_ vs HR_mPFS_ [McNemar’s test p-value = 0.014]).

In comparisons testing ICI plus chemotherapy, a fair agreement was observed between HR_OS_ and all the surrogate measures (Cohen’s Kappa coefficient was 0.37 [95% CI: 0.04, 0.71] and McNemar’s test p-value 0.014 for HR_OS_ vs HR_PFS_, rRMST_PFS_ and rRMST_mPFS_). In ICI plus ICI or other treatment(s) strata, the agreement was very poor with all the surrogate measures (Cohen’s Kappa coefficient ranged from -0.06 for HR_OS_ vs HR_mPFS_ [McNemar’s test p-value = 0.257] to 0.12 for HR_OS_ vs HR_PFS_ and rRMST_PFS_ [McNemar’s test p-value = 0.414]).

**Figure 2:**
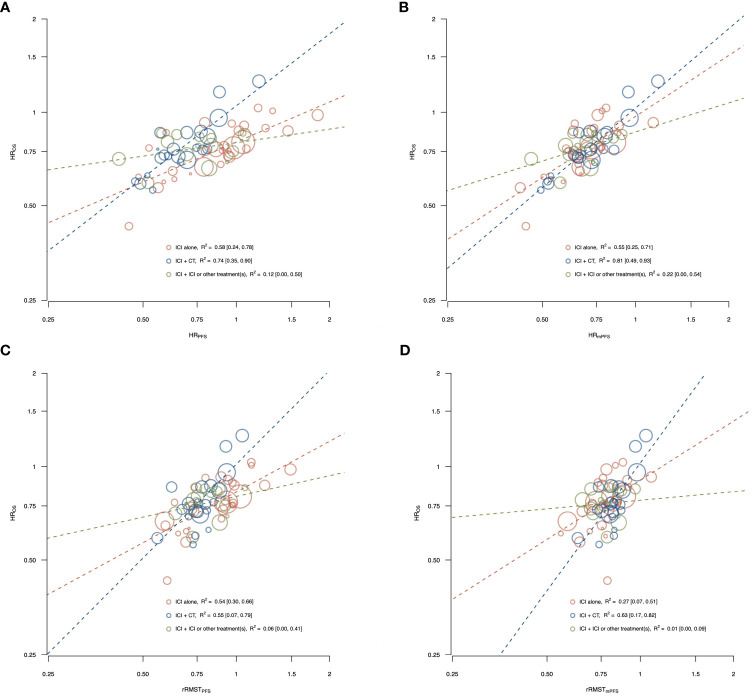
Correlations between effects of ICIs on OS and the potential surrogate endpoints, PFS **(A, C)** and mPFS **(B, D)** by type of treatment administered in the experimental arm. The figure shows the correlations between effects of ICIs on OS and the potential surrogate endpoints, PFS **(A, C)** and mPFS **(B, D)** according to the type of treatment administered in the experimental arm [i.e., ICI alone, ICI plus chemotherapy, and ICI plus ICI or other treatment(s)]. The treatment effects are measured by the HR for OS, and by the HR and the rRMST for the two surrogate endpoints. Each circle represents a comparison, and the surface area of the circle is proportional to the number of patients in the corresponding comparison. Red circles represent comparisons with ICI alone as experimental arm, blue circles represent comparisons with ICI plus chemotherapy as experimental arm, and green circles represent comparisons with ICI plus ICI or other treatment(s) as experimental arm. Dashed lines represent weighted regression lines. The R^2^ coefficients, with their 95% CI (displayed in square brackets), were reported in the legend.

**Figure 2** shows the correlations between effects of ICIs on OS (y-axis) and the potential surrogate endpoints (x-axes; i.e., panel A: HR_PFS_, panel B: HR_mPFS_, panel C: rRMST_PFS_, and panel D: rRMST_mPFS_) according to the type of treatment administered in the experimental arm.

Among comparisons testing ICI alone, a moderate association with HR_OS_ was observed for HR_PFS_ (R^2^ = 0.58, 95% CI: 0.24, 0.78), HR_mPFS_ (R^2^ = 0.55, 95% CI: 0.25, 0.71) and rRMST_PFS_ (R^2^ = 0.54, 95% CI: 0.30, 0.66), while for rRMST_mPFS_ the correlation was weak (R^2^ = 0.27, 95% CI: 0.07, 0.51).

Among comparisons with ICI plus chemotherapy as experimental arm, a strong association with HR_OS_ was observed for HR_PFS_ (R^2^ = 0.74, 95% CI: 0.35, 0.90) and HR_mPFS_ (R^2^ = 0.81, 95% CI: 0.49, 0.93), while the R^2^ coefficient was 0.55 (95% CI: 0.07, 0.79) for rRMST_PFS_ and 0.63 (95% CI: 0.17, 0.82) for rRMST_mPFS_.

In the ICI plus ICI or other treatment(s) strata, the associations were very weak for all the surrogate endpoints and treatment effect measures with the R^2^ ranging from 0.01 to 0.22.

**Figure 3:**
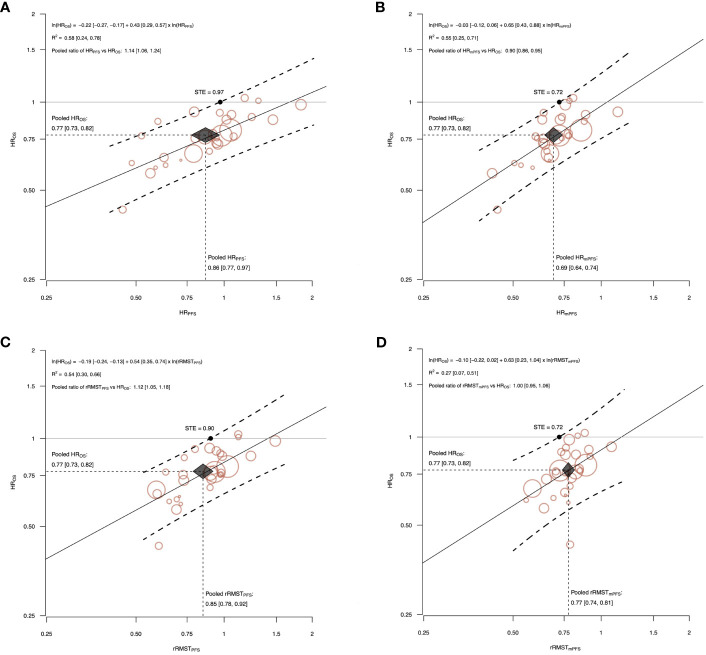
The correlations between effects of ICI alone on OS and the potential surrogate endpoints, PFS **(A, C)** and mPFS **(B, D)**. The figure shows the correlations between effects of ICI alone on OS and the potential surrogate endpoints, PFS **(A, C)** and mPFS **(B, D)**. The treatment effects are measured by HR for OS, and by the HR and the rRMST for the two surrogate endpoints. Each circle represents a comparison, and the surface area of the circle is proportional to the number of patients in the corresponding comparison. Straight line represents weighted regression line. Dashed lines represent 95% prediction bands based on the values predicted by the weighted regression model. The surrogate threshold effect (STE) is represented by the intersection point between the horizonal line y=1 and the upper 95% prediction band. Black diamond indicates the meta-analytic pooled estimate. The diamond’s width represents the 95% CI of the surrogate pooled estimate, and height represents the 95% CI of the HR_OS_ pooled estimate. The surrogacy equation between the log-transformed treatment effects and the ln-HR_OS_ estimated from the weighted linear regression, the R^2^ coefficient, and the pooled ratio between surrogate endpoint and HR_OS_ were also reported with their 95% CI (displayed in square brackets).

The surrogacy equations between the log-transformed treatment effects and the ln-HR_OS_ estimated from the WLS regression, along with the R^2^ coefficient, the prediction bands, and STE were displayed in **Figure 3**, **Supplementary Figures S3** and **S4** for ICI alone, ICI plus chemotherapy, and ICI plus ICI or other treatment(s), respectively.

The slope of the surrogacy WLS regression lines significantly differed from zero for all the analysis on ICI alone and ICI plus chemotherapy comparisons.

## Discussion

There is controversy on the value of PFS as surrogate endpoint for OS in RCTs testing anticancer immunotherapy in patients with advanced solid tumors (2, 3, 79, 80). Another issue of intense debate is the most adequate metric to capture the treatment effect on PFS, since the widely adopted HR relies on the PH assumption, which is frequently violated in immunotherapy trials (4).

To our knowledge, we reported here the most comprehensive and updated analysis exploring these two related issues: i) the value of the PFS as surrogate endpoint for OS in trials testing ICIs, as compared with the alternative endpoint represented by mPFS; ii) the suitability of the HR to measure the treatment effect on PFS, as compared with the alternative metric represented by RMST.

Our findings tried to increase the understanding of the afore-mentioned conflicting results regarding the surrogacy value of PFS (2, 3, 79, 80). When all the RCTs were pooled together, none of the endpoints (i.e., PFS and mPFS) or metrics (i.e., HR and RMST) investigated had a strong association with HR_OS._

For example, we found that the R^2^ of the association between HR_PFS_ and HR_OS_ was 0.38 (95% CI: 0.23, 0.52). This means that only 38% of the variability among treatment effects on OS is explained by the effects observed on PFS, far from the R^2^ cut-off value of 0.70, which is considered optimal for a candidate surrogate endpoint by international guidelines (8).

However, when comparisons testing different types of treatments were analyzed separately, we observed a moderate (i.e., 0.5≤R^2^ ≤ 0.69) or a strong (i.e., R^2^≥0.7) association between HR_PFS_ and HR_OS_ in the two groups of comparisons testing ICI alone or combined with chemotherapy, respectively.

This discrepancy between results of overall and stratified analyses might be explained by the fact that the HR_PFS_ and HR_OS_ estimates were well aligned within the two groups, but along two different regression lines. The regression line fitting the HR_PFS_ over HR_OS_ in comparisons testing ICI alone had lower values of both the intercept (-0.22) and the slope (0.43), compared to those estimated in the ICI plus chemotherapy group (intercept=0.05, slope=0.77).

The slope indicates the steepness of the regression line and captures the average rate of HR_OS_ change when HR_PFS_ increases by 1 unit. Slope values near zero indicate that negligible HR_OS_ changes occur as the HR_PFS_ changes, while slope values that are progressively further from zero indicate rates of HR_OS_ change increasingly larger. Our results suggests that in both groups of trials an improvement of PFS favoring the experimental arm translated into an OS improvement, but the amount of OS gain with the same treatment effect on PFS was different. As a matter of fact, even if the pooled HR_OS_ was very similar in the two groups (0.77 [95% CI: 0.73, 0.82] and 0.78 [95% CI: 0.73, 0.84] for ICI alone or combined with chemotherapy, respectively), the pooled HR_PFS_ resulted significantly heterogeneous (0.86 [95% CI: 0.77, 0.97] and 0.68 [95% CI: 0.62, 0.74], respectively). It might be explained by the fact that the biological effects and the impact on the natural history of disease of each type of treatment can differ substantially, and thus the amount of PFS improvement that eventually translates into an effect on OS is strictly treatment-dependent.

Homogeneity of treatment types and their biological mechanisms of action should be guaranteed in surrogacy analyses (8). If this principle is overlooked, even a strong association between the candidate surrogate and the true endpoint can be hidden, leading to the erroneous conclusion of absence of surrogacy.

Consistently, the absence of PFS surrogacy observed in the third group of comparisons [i.e., ICI plus ICI or other treatment(s)] can be attributed to the heterogeneous spectrum of therapies considered, including the combination of ICI with anti-angiogenesis agents, targeted therapies, or different types of ICIs. The limited number of RCTs available for each of these treatment types precluded the possibility to perform more specific analyses. Therefore, no conclusions can be drawn on the surrogacy value of PFS for immunotherapy strategies other than ICI alone or combined with chemotherapy.

Finally, our results showed that mPFS did not confer an advantage over PFS.

Concerning the issue of the best metric for measuring the treatment effect on PFS, it has been reported that the RMST can provide theoretical advantages over the HR. RMST is not affected by deviations from the PH assumption, which is common in immunotherapy trials due to the intrinsic mechanisms of action of ICIs that lead to delayed responses and long-term responders (4, 81).

Although in 54% of included comparisons (36 out of 67) the PH assumption was violated (for PFS or OS only, or both), we unexpectedly found that the HR outperformed the rRMST, yielding higher R^2^ values for PFS over OS in all of the explored contexts. Notably, despite the fact that the largest difference favoring HR versus rRMST was observed in the group of trials testing ICI plus chemotherapy, where the PH assumption held in the majority of cases for both OS and PFS, no advantage for rRMST was observed in the other two groups of comparisons, where the PH assumption was largely violated.

Our analysis revealed that HR_PFS_ is limited by the tendency to significantly underestimate the treatment effect size observed on HR_OS_ in comparisons testing ICI alone (pooled ratio HR_PFS_/HR_OS_=1.14, 95% CI: 1.06, 1.24) and, on the contrary, to significantly overestimate it in comparisons testing the combination of ICI plus chemotherapy (pooled ratio HR_PFS_/HR_OS_=0.88, 95% CI: 0.83, 0.93).

This had in turn relevant impact on the STE of HR_PFS_, which is defined as the minimum value of the HR_PFS_ necessary to be observed in a future RCT to confidently predict an OS benefit. Our results showed that a HR_PFS_ lower than 1 could be enough to predict a significant OS benefit in RCTs testing ICI alone, while a HR_PFS_ lower than 0.70 is required in trials testing ICI plus chemotherapy.

The overestimation of the final effect on HR_OS_ by HR_PFS_ is expected, since all the events considered in the OS endpoint are also included in the PFS. Similar findings were previously reported in surrogacy analyses on RCTs testing chemotherapy or targeted therapy (80, 82). In contrast, the underestimation of the final effect on HR_OS_ by HR_PFS_ observed in trials testing ICI alone is quite unusual and it could be due to the pseudo-progression events. These events are specifically observed in patients treated with ICI alone and they can lead to an erroneous and systematic underestimation of the ICI effect on PFS without affecting patients’ OS. In accordance with this hypothesis, the underestimation of the treatment effect on OS in comparisons testing ICI alone was not observed when considering the mPFS that accounts for the pseudo-progression events. However, the weak value of the R^2^ precluded the possibility to use such endpoint in this context.

It’s worth noting that the incidence of pseudo-progression events has been reported to be higher among patients treated with anti-CTLA4 monotherapy as compared to anti-PD(L)1 monotherapy. Consequently, the surrogacy value of mPFS might be substantially higher in trials exclusively testing anti-CTLA4 treatments. However, the limited numbers of RCTs assessing anti-CTLA4 monotherapy precluded conducting a more detailed analysis.

The immune-related RECIST (iRECIST) are new response criteria specifically designed to assess the activity of immunotherapy, and to correctly categorize real versus pseudo-progression events (83). The adoption of iRECIST to categorize the events included in the PFS would probably improve its tendency to underestimate treatment effects on OS in trials testing ICI alone, but additional study and validation of iRECIST are required.

Our study has several strengths. It is the most comprehensive and updated analysis on such relevant topic, and it includes a large number of RCTs and patients. The efforts to reconstruct the IPD of more than 36,000 patients using a validated algorithm allowed to reliably assess and compare different endpoints and metrics. Also, the wide range of treatment effect estimates reported in the included trials for both PFS and OS contributed to ensuring adequate generalizability of the analysis.

The main limitation of our analysis is that it is based on reconstructed rather than original IPD. Original IPD allow for checking the plausibility of randomization sequences, verifying data integrity and consistency, fitting bivariate and copula-based models, which are among the preferred methods of assessing trial-level associations, adjusting the analyses for baseline prognostic covariates, and accounting for the fact that each within-trial surrogate outcome is estimated with error (84). Nevertheless, the specific goal of our analysis was to assess surrogacy at trial-level, and we used only data from high-quality RCTs. Therefore, an analysis based on original IPD is unlikely to substantially change our conclusions.

Furthermore, results from the analysis stratified by type of treatment administrated in the experimental arm deserve further validation to be considered conclusive. Finally, it could be possible that the shape of the curves for PFS and thus its surrogacy value could meaningfully be affected by specific inclusion criteria used to select patients populations enrolled in RCTs, especially the enrichment for molecular and clinical biomarkers predictive of response to immunotherapy, such as expression levels of PDL1, tumor mutational burden, smoking habits and gender. The lack of original IPD precluded conducting such types of granular analyses.

A relevant consideration should be highlighted. Regulatory agencies consider the HR_OS_ as the gold-standard measure for assessing treatment effects in RCTs and approving new drugs (85). As a result, we used it as the reference measure in our surrogacy analysis. In conclusion, our results showed that HR_PFS_ had a strong surrogacy value for HR_OS_ in comparisons testing ICI in combination with chemotherapy and moderate in comparisons testing ICI alone. Therefore, it should remain the reference surrogate endpoint in such contexts. Even in the presence of significant deviation from the PH assumption, the available evidence does not support the use of alternative endpoints, such as the mPFS, or metrics, such as the RMST.

Finally, two caveats should be highlighted. First, the available evidence does not allow for an adequate investigation of the value of HR_PFS_ as surrogate for other types of immunotherapy treatment strategies. Second, when using treatment effects on HR_PFS_ to predict those on HR_OS_, the tendency to either underestimate or overestimate the final OS should be taken into account, depending on the type of treatment under investigation.

## Author’s note

The lead authors (VB and FC) affirm that this manuscript is an honest, accurate, and transparent account of the study being reported; that no important aspects of the study have been omitted; and that any discrepancies from the study as planned have been explained.

## Data availability statement

The original contributions presented in the study are included in the article/**Supplementary Material**. Further inquiries can be directed to the corresponding author.

## Author contributions

IS: Conceptualization, Data curation, Formal Analysis, Investigation, Methodology, Project administration, Resources, Supervision, Validation, Visualization, Writing – original draft, Writing – review & editing. EP: Data curation, Formal Analysis, Investigation, Methodology, Project administration, Writing – review & editing. LP: Conceptualization, Investigation, Supervision, Visualization, Writing – review & editing. CO: Data curation, Formal Analysis, Investigation, Methodology, Validation, Visualization, Writing – review & editing. MM: Data curation, Formal Analysis, Validation, Visualization, Writing – review & editing. CS: Conceptualization, Data curation, Formal Analysis, Investigation, Methodology, Supervision, Validation, Visualization, Writing – review & editing. TP: Supervision, Validation, Visualization, Writing – review & editing. JC: Conceptualization, Investigation, Methodology, Supervision, Validation, Visualization, Writing – review & editing. GG: Conceptualization, Investigation, Methodology, Supervision, Validation, Visualization, Writing – review & editing. MP: Investigation, Methodology, Supervision, Validation, Visualization, Writing – review & editing. RG: Conceptualization, Formal Analysis, Investigation, Methodology, Project administration, Supervision, Validation, Visualization, Writing – review & editing. VB: Conceptualization, Data curation, Formal Analysis, Funding acquisition, Investigation, Methodology, Project administration, Resources, Software, Supervision, Validation, Visualization, Writing – original draft, Writing – review & editing. FC: Conceptualization, Data curation, Investigation, Methodology, Project administration, Resources, Supervision, Validation, Visualization, Writing – review & editing.

## References

[1] Lenzer J Brownlee S . Should regulatory authorities approve drugs based on surrogate endpoints? BMJ (2021) 374:n2059. doi: 10.1136/bmj.n2059 34526303

[2] Mushti SL Mulkey F Sridhara R . Evaluation of overall response rate and progression-free survival as potential surrogate endpoints for overall survival in immunotherapy trials. Clin Cancer Res (2018) 24:2268–75. doi: 10.1158/1078-0432.CCR-17-1902 29326281

[3] Nie RC Chen FP Yuan SQ Luo YS Chen S Chen YM . Evaluation of objective response, disease control and progression-free survival as surrogate end-points for overall survival in anti–programmed death-1 and anti–programmed death ligand 1 trials. Eur J Cancer (2019) 106:1–11. doi: 10.1016/j.ejca.2018.10.011 30453169

[4] Alexander BM Schoenfeld JD Trippa L . Hazards of hazard ratios — Deviations from model assumptions in immunotherapy. N Engl J Med (2018) 378:1158–9. doi: 10.1056/NEJMc1716612 29562148

[5] Royston P Parmar MKB . Restricted mean survival time: an alternative to the hazard ratio for the design and analysis of randomized trials with a time-to-event outcome. BMC Med Res Methodol (2013) 13:152. doi: 10.1186/1471-2288-13-152 24314264 PMC3922847

[6] Wang ZX Wu HX Xie L Lin WH Liang F Li J . Exploration of modified progression-free survival as a novel surrogate endpoint for overall survival in immuno-oncology trials. J Immunother Cancer (2021) 9:e002114. doi: 10.1136/jitc-2020-002114 33795385 PMC8021890

[7] Moher D Liberati A Tetzlaff J Altman DG . Preferred reporting items for systematic reviews and meta-analyses: the PRISMA statement. BMJ (2009) 339:b2535. 19622551 10.1136/bmj.b2535 PMC2714657

[8] Xie W Halabi S Tierney JF Sydes MR Collette L Dignam JJ . A systematic review and recommendation for reporting of surrogate endpoint evaluation using meta-analyses. JNCI Cancer Spectr (2019) 3:pkz002. doi: 10.1093/jncics/pkz002 31360890 PMC6649812

[9] Higgins JPT Altman DG Sterne JAC . Chapter 8: Assessing risk of bias in included studies. In: Higgins JPT Churchill R Chandler J Cumpston MS , editors. Cochrane Handbook for Systematic Reviews of Interventions version 5.2.0 (updated June 2017). Cochrane (2017). Available at: https://training.cochrane.org/handbook.

[10] Rohatgi A . WebPlotDigitizer user manual version 4.3 (2020). Available at: https://automeris.io/WebPlotDigitizer/userManual.pdf.

[11] Guyot P Ades AE Ouwens MJNM Welton NJ . Enhanced secondary analysis of survival data: reconstructing the data from published Kaplan-Meier survival curves. BMC Med Res Methodol (2012) 12:9. doi: 10.1186/1471-2288-12-9 22297116 PMC3313891

[12] Robert C Thomas L Bondarenko I O'Day S Weber J Garbe C . Ipilimumab plus dacarbazine for previously untreated metastatic melanoma. N Engl J Med (2011) 364:2517–26. doi: 10.1056/NEJMoa1104621 21639810

[13] Reck M Luft A Szczesna A Havel L Kim SW Akerley W . Phase III randomized trial of ipilimumab plus etoposide and platinum versus placebo plus etoposide and platinum in extensive-stage small-cell lung cancer. J Clin Oncol (2016) 34:3740–8. doi: 10.1200/JCO.2016.67.6601 27458307

[14] Fehrenbacher L von Pawel J Park K Rittmeyer A Gandara DR Ponce Aix S . Updated efficacy analysis including secondary population results for OAK: A randomized phase III study of atezolizumab versus docetaxel in patients with previously treated advanced non-small cell lung cancer. J Thorac Oncol (2018) 13:1156–70. doi: 10.1016/j.jtho.2018.04.039 29777823

[15] Awad MM Gadgeel SM Borghaei H Patnaik A Yang JC Powell SF . Long-term overall survival from KEYNOTE-021 cohort G: pemetrexed and carboplatin with or without pembrolizumab as first-line therapy for advanced nonsquamous NSCLC. J Thorac Oncol (2021) 16:162–8. doi: 10.1016/j.jtho.2020.09.015 33069888

[16] Carbone DP Reck M Paz-Ares L Creelan B Horn L Steins M . First-line nivolumab in stage IV or recurrent non–small-cell lung cancer. N Engl J Med (2017) 376:2415–26. doi: 10.1056/NEJMoa1613493 PMC6487310 28636851

[17] Ferris RL Blumenschein G Fayette J Guigay J Colevas AD Licitra L . Nivolumab for recurrent squamous-cell carcinoma of the head and neck. N Engl J Med (2016) 375:1856–67. doi: 10.1056/NEJMoa1602252 PMC5564292 27718784

[18] Reck M Rodriguez-Abreu D Robinson AG Hui R Csőszi T Fülöp A . Pembrolizumab versus chemotherapy for PD-L1-positive non-small-cell lung cancer. N Engl J Med (2016) 375:1823–33. doi: 10.1056/NEJMoa1606774 27718847

[19] Reck M Rodríguez-Abreu D Robinson AG Hui R Csőszi T Fülöp A . Updated analysis of KEYNOTE-024: Pembrolizumab versus platinum-based chemotherapy for advanced non–small-cell lung cancer with PD-L1 tumor proportion score of 50% or greater. J Clin Oncol (2019) 37:537–46. doi: 10.1200/JCO.18.00149 30620668

[20] Mok TSK Wu YL Kudaba I Kowalski DM Cho BC Turna HZ . Pembrolizumab versus chemotherapy for previously untreated, PD-L1-expressing, locally advanced or metastatic non-small-cell lung cancer (KEYNOTE-042): a randomised, open-label, controlled, phase 3 trial. Lancet (2019) 393:1819–30. doi: 10.1016/S0140-6736(18)32409-7 30955977

[21] Motzer RJ Tannir NM McDermott DF Arén Frontera O Melichar B Choueiri TK . Nivolumab plus Ipilimumab versus Sunitinib in Advanced Renal-Cell Carcinoma. N Engl J Med (2018) 378:1277–90. doi: 10.1056/NEJMoa1712126 PMC5972549 29562145

[22] Cohen EEW Soulières D Le Tourneau C Dinis J Licitra L Ahn MJ . Pembrolizumab versus methotrexate, docetaxel, or cetuximab for recurrent or metastatic head-and-neck squamous cell carcinoma (KEYNOTE-040): a randomised, open-label, phase 3 study. Lancet (2019) 393:156–67. doi: 10.1016/S0140-6736(18)31999-8 30509740

[23] Bellmunt J de Wit R Vaughn DJ Fradet Y Lee JL Fong L . Pembrolizumab as second-line therapy for advanced urothelial carcinoma. N Engl J Med (2017) 376:1015–26. doi: 10.1056/NEJMoa1613683 PMC5635424 28212060

[24] Borghaei H Gettinger S Vokes EE Chow LQM Burgio MA de Castro Carpeno J . Five-year outcomes from the randomized , phase III trials checkMate 017 and 057: nivolumab versus docetaxel in previously treated non–small-cell lung cancer. J Clin Oncol (2021) 39:723–33. doi: 10.1200/JCO.20.01605 PMC8078445 33449799

[25] Chen LT Satoh T Ryu MH Chao Y Kato K Chung HC . A phase 3 study of nivolumab in previously treated advanced gastric or gastroesophageal junction cancer (ATTRACTION-2): 2-year update data. Gastric Cancer (2020) 23:510–9. doi: 10.1007/s10120-019-01034-7 PMC7165140 31863227

[26] Planchard D Reinmuth N Orlov S Fischer JR Sugawara S Mandziuk S . ARCTIC: durvalumab with or without tremelimumab as third-line or later treatment of metastatic non-small-cell lung cancer. Ann Oncol (2020) 31:609–18. doi: 10.1016/j.annonc.2020.02.006 32201234

[27] Socinski MA Jotte RM Cappuzzo F Orlandi F Stroyakovskiy D Nogami N . Atezolizumab for first-line treatment of metastatic nonsquamous NSCLC. N Engl J Med (2018) 378:2288–301. doi: 10.1056/NEJMoa1716948 29863955

[28] Socinski MA Nishio M Jotte RM Cappuzzo F Orlandi F Stroyakovskiy D . IMpower150 final overall survival analyses for atezolizumab plus bevacizumab and chemotherapy in first-line metastatic nonsquamous NSCLC. J Thorac Oncol (2021) 16:1909–24. doi: 10.1016/j.jtho.2021.07.009 34311108

[29] West H McCleod M Hussein M Morabito A Rittmeyer A Conter HJ . Atezolizumab in combination with carboplatin plus nab-paclitaxel chemotherapy compared with chemotherapy alone as first-line treatment for metastatic non-squamous non-small-cell lung cancer (IMpower130): a multicentre, randomised, open-label, phase 3 tria. Lancet Oncol (2019) 20:924–37. doi: 10.1016/S1470-2045(19)30167-6 31122901

[30] Jotte R Cappuzzo F Vynnychenko I Stroyakovskiy D Rodríguez-Abreu D Hussein M . Atezolizumab in combination with carboplatin and nab-paclitaxel in advanced squamous NSCLC (IMpower131): results from a randomized phase III trial. J Thorac Oncol (2020) 15:1351–60. doi: 10.1016/j.jtho.2020.03.028 32302702

[31] Barlesi F Vansteenkiste J Spigel D Ishii H Garassino M de Marinis F . Avelumab versus docetaxel in patients with platinum-treated advanced non-small-cell lung cancer (JAVELIN Lung 200): an open-label, randomised, phase 3 study. Lancet Oncol (2018) 19:1468–79. doi: 10.1016/S1470-2045(18)30673-9 30262187

[32] Park K Özgüroğlu M Vansteenkiste J Spigel D Yang JCH Ishii H . Avelumab versus docetaxel in patients with platinum-treated advanced NSCLC: 2-year follow-up from the JAVELIN lung 200 phase 3 trial. J Thorac Oncol (2021) 16:1369–78. doi: 10.1016/j.jtho.2021.03.009 33845211

[33] Herbst RS Giaccone G de Marinis F Reinmuth N Vergnenegre A Barrios CH . Atezolizumab for first-line treatment of PD-L1–selected patients with NSCLC. N Engl J Med (2020) 383:1328–39. doi: 10.1056/NEJMoa1917346 32997907

[34] Emens LA Adams S Barrios CH Diéras V Iwata H Loi S . First-line atezolizumab plus nab-paclitaxel for unresectable, locally advanced, or metastatic triple-negative breast cancer: IMpassion130 final overall survival analysis. Ann Oncol (2021) 32:983–93. doi: 10.1016/j.annonc.2021.05.355 34272041

[35] Motzer RJ Escudier B McDermott DF George S Hammers HJ Srinivas S . Nivolumab versus everolimus in advanced renal-cell carcinoma. N Engl J Med (2015) 373:1803–13. doi: 10.1056/NEJMoa1510665 PMC5719487 26406148

[36] Rizvi NA Cho BC Reinmuth N Lee KH Luft A Ahn MJ . Durvalumab with or without tremelimumab vs standard chemotherapy in first-line treatment of metastatic non-small cell lung cancer: the MYSTIC phase 3 randomized clinical trial. JAMA Oncol (2020) 6:661–74. doi: 10.1001/jamaoncol.2020.0237 PMC7146551 32271377

[37] Hellmann MD Paz-Ares L Bernabe Caro R Zurawski B Kim SW Carcereny Costa E . Nivolumab plus ipilimumab in advanced non–small-cell lung cancer. N Engl J Med (2019) 381:2020–31. doi: 10.1056/NEJMoa1910231 31562796

[38] Paz-Ares LG Ciuleanu TE Lee JS Urban L Bernabe Caro R Park K . Nivolumab (NIVO) plus ipilimumab (IPI) versus chemotherapy (chemo) as first-line (1L) treatment for advanced non-small cell lung cancer (NSCLC): 4-year update from CheckMate 227. J Clin Oncol (2021) 39:9016. doi: 10.1200/JCO.2021.39.15_suppl.9016

[39] Spigel DR Vicente D Ciuleanu TE Gettinger S Peters S Horn L . Second-line nivolumab in relapsed small-cell lung cancer: CheckMate 331. Ann Oncol (2021) 32:631–41. doi: 10.1016/j.annonc.2021.01.071 33539946

[40] Powles T van der Heijden MS Castellano D Galsky MD Loriot Y . Durvalumab alone and durvalumab plus tremelimumab versus chemotherapy in previously untreated patients with unresectable, locally advanced or metastatic urothelial carcinoma (DANUBE): a randomised, open-label, multicentre, phase 3 trial. Lancet Oncol (2020) 21:1574–88. doi: 10.1016/S1470-2045(20)30541-6 32971005

[41] Winer EP Lipatov O Im SA Goncalves A Muñoz-Couselo E Lee KS . Pembrolizumab versus investigator-choice chemotherapy for metastatic triple-negative breast cancer (KEYNOTE-119): a randomised, open-label, phase 3 trial. Lancet Oncol (2021) 22:499–511. doi: 10.1016/S1470-2045(20)30754-3 33676601

[42] Kato K Cho BC Takahashi M Okada M Lin CY Chin K . Nivolumab versus chemotherapy in patients with advanced oesophageal squamous cell carcinoma refractory or intolerant to previous chemotherapy (ATTRACTION-3): a multicentre, randomised, open-label, phase 3 trial. Lancet Oncol (2019) 20:1506–17. doi: 10.1016/S1470-2045(19)30626-6 31582355

[43] Rodríguez-Abreu D Powell SF Hochmair MJ Gadgeel S Esteban E Felip E . Pemetrexed plus platinum with or without pembrolizumab in patients with previously untreated metastatic nonsquamous NSCLC: protocol-specified final analysis from KEYNOTE-189. Ann Oncol (2021) 32:881–95. doi: 10.1016/j.annonc.2021.04.008 33894335

[44] Lu S Wang J Cheng Y Mok T Chang J Zhang L . Nivolumab versus docetaxel in a predominantly Chinese patient population with previously treated advanced non-small cell lung cancer: 2-year follow-up from a randomized, open-label, phase 3 study (CheckMate 078). Lung Cancer (2021) 152:7–14. doi: 10.1016/j.lungcan.2020.11.013 33321441

[45] Moehler M Dvorkin M Boku N Özgüroğlu M Ryu MH Muntean AS . Phase III trial of avelumab maintenance after first-line induction chemotherapy versus continuation of chemotherapy in patients with gastric cancers: results from JAVELIN gastric 100. J Clin Oncol (2021) 39:966–77. doi: 10.1200/JCO.20.00892 PMC8078426 33197226

[46] Larkin J Minor D D’Angelo S Neyns B Smylie M Miller WH Jr . Overall survival in patients with advanced melanoma who received nivolumab versus investigator’s choice chemotherapy in checkMate 037: A randomized, controlled, open-label phase III trial. J Clin Oncol (2018) 36:383–90. doi: 10.1200/JCO.2016.71.8023 PMC6804912 28671856

[47] Nishio M Barlesi F West H Ball S Bordoni R Cobo M . Atezolizumab plus chemotherapy for first-line treatment of nonsquamous NSCLC: results from the randomized phase 3 IMpower132 trial. J Thorac Oncol (2021) 16:653–64. doi: 10.1016/j.jtho.2020.11.025 33333328

[48] Choueiri TK Motzer RJ Rini BI Haanen J Campbell MT Venugopal B . Updated efficacy results from the JAVELIN Renal 101 trial: first-line avelumab plus axitinib versus sunitinib in patients with advanced renal cell carcinoma. Ann Oncol (2020) 31:1030–9. doi: 10.1016/j.annonc.2020.04.010 PMC8436592 32339648

[49] Horn L Mansfield AS Szczęsna A Havel L Krzakowski M Hochmair MJ . First-line atezolizumab plus chemotherapy in extensive-stage small-cell lung cancer. N Engl J Med (2018) 379:2220–9. doi: 10.1056/NEJMoa1809064 30280641

[50] Liu SV Reck M Mansfield AS Mok T Scherpereel A Reinmuth N . Updated overall survival and PD-L1 subgroup analysis of patients with extensive-stage small-cell lung cancer treated with atezolizumab, carboplatin, and etoposide (IMpower133). J Clin Oncol (2021) 39:619–30. doi: 10.1200/JCO.20.01055 PMC8078320 33439693

[51] Paz-Ares L Vicente D Tafreshi A Robinson A Soto Parra H Mazières J . A randomized, placebo-controlled trial of pembrolizumab plus chemotherapy in patients with metastatic squamous NSCLC: protocol-specified final analysis of KEYNOTE-407. J Thorac Oncol (2020) 15:1657–69. doi: 10.1016/j.jtho.2020.06.015 32599071

[52] Galsky MD Arija JÁA Bamias A Davis ID De Santis M Kikuchi E . Atezolizumab with or without chemotherapy in metastatic urothelial cancer (IMvigor130): a multicentre, randomised, placebo-controlled phase 3 trial. Lancet (2020) 395:1547–57. doi: 10.1016/S0140-6736(20)30230-0 32416780

[53] Motzer R Alekseev B Rha SY Porta C Eto M Powles T . Lenvatinib plus pembrolizumab or everolimus for advanced renal cell carcinoma. N Engl J Med (2021) 384:1289–300. doi: 10.1056/NEJMoa2035716 33616314

[54] Powles T Csőszi T Özgüroğlu M Matsubara N Géczi L Cheng SY . Pembrolizumab alone or combined with chemotherapy versus chemotherapy as first-line therapy for advanced urothelial carcinoma (KEYNOTE-361): a randomised, open-label, phase 3 trial. Lancet Oncol (2021) 22:931–45. doi: 10.1016/S1470-2045(21)00152-2 34051178

[55] Chen EX Jonker DJ Loree JM Kennecke HF Berry SR Couture F . Effect of combined immune checkpoint inhibition vs best supportive care alone in patients with advanced colorectal cancer: the canadian cancer trials group CO.26 study. JAMA Oncol (2020) 6:831–8. doi: 10.1001/jamaoncol.2020.0910 PMC7206536 32379280

[56] Janjigian YY Shitara K Moehler M Garrido M Salman P Shen L . First-line nivolumab plus chemotherapy versus chemotherapy alone for advanced gastric, gastro-oesophageal junction, and oesophageal adenocarcinoma (CheckMate 649): a randomised, open-label, phase 3 trial. Lancet (2021) 398:27–40. doi: 10.1016/S0140-6736(21)00797-2 34102137 PMC8436782

[57] Robert C Long GV Brady B Dutriaux C Maio M Mortier L . Nivolumab in previously untreated melanoma without BRAF mutation. N Engl J Med (2015) 372:320–30. doi: 10.1056/NEJMoa1412082 25399552

[58] Baas P Scherpereel A Nowak AK Fujimoto N Peters S Tsao AS . First-line nivolumab plus ipilimumab in unresectable Malignant pleural mesothelioma (CheckMate 743): a multicentre, randomised, open-label, phase 3 trial. Lancet (2021) 397:375–86. doi: 10.1016/S0140-6736(20)32714-8 33485464

[59] Gutzmer R Stroyakovskiy D Gogas H Robert C Lewis K Protsenko S . Atezolizumab, vemurafenib, and cobimetinib as first-line treatment for unresectable advanced BRAFV600 mutation-positive melanoma (IMspire150): primary analysis of the randomised, double-blind, placebo-controlled, phase 3 trial. Lancet (2020) 395:1835–44. doi: 10.1016/S0140-6736(20)30934-X 32534646

[60] Lee NY Ferris RL Psyrri A Haddad RI Tahara M Bourhis J . Avelumab plus standard-of-care chemoradiotherapy versus chemoradiotherapy alone in patients with locally advanced squamous cell carcinoma of the head and neck: a randomised, double-blind, placebo-controlled, multicentre, phase 3 trial. Lancet Oncol (2021) 22:450–62. doi: 10.1016/S1470-2045(20)30737-3 33794205

[61] Goldman JW Dvorkin M Chen Y Reinmuth N Hotta K Trukhin D . Durvalumab, with or without tremelimumab, plus platinum–etoposide versus platinum–etoposide alone in first-line treatment of extensive-stage small-cell lung cancer (CASPIAN): updated results from a randomised, controlled, open-label, phase 3 trial. Lancet Oncol (2021) 22:51–65. doi: 10.1016/S1470-2045(20)30539-8 33285097

[62] Sezer A Kilickap S Gümüş M Bondarenko I Özgüroğlu M Gogishvili M . Cemiplimab monotherapy for first-line treatment of advanced non-small-cell lung cancer with PD-L1 of at least 50%: a multicentre, open-label, global, phase 3, randomised, controlled trial. Lancet (2021) 397:592–604. doi: 10.1016/S0140-6736(21)00228-2 33581821

[63] Sugawara S Lee JS Kang JH Kim HR Inui N Hida T . Nivolumab with carboplatin, paclitaxel, and bevacizumab for first-line treatment of advanced nonsquamous non-small-cell lung cancer. Ann Oncol (2021) 32:1137–47. doi: 10.1016/j.annonc.2021.06.004 34139272

[64] Miles D Gligorov J André F Cameron D Schneeweiss A Barrios C . Primary results from IMpassion131, a double-blind, placebo-controlled, randomised phase III trial of first-line paclitaxel with or without atezolizumab for unresectable locally advanced/metastatic triple-negative breast cancer. Ann Oncol (2021) 32:994–1004. doi: 10.1016/j.annonc.2021.05.801 34219000

[65] Zhou C Chen G Huang Y Zhou J Lin L Feng J . Camrelizumab plus carboplatin and pemetrexed versus chemotherapy alone in chemotherapy-naive patients with advanced non-squamous non-small-cell lung cancer (CameL): a randomised, open-label, multicentre, phase 3 trial. Lancet Respir Med (2021) 9:305–14. doi: 10.1016/S2213-2600(20)30365-9 33347829

[66] Choueiri TK Powles T Burotto M Escudier B Bourlon MT Zurawski B . Nivolumab plus Cabozantinib versus Sunitinib for Advanced Renal-Cell Carcinoma. N Engl J Med (2021) 384:829–41. doi: 10.1056/NEJMoa2026982 PMC8436591 33657295

[67] Sun JM Shen L Shah MA Enzinger P Adenis A Doi T . Pembrolizumab plus chemotherapy versus chemotherapy alone for first-line treatment of advanced oesophageal cancer (KEYNOTE-590): a randomised, placebo-controlled, phase 3 study. Lancet (2021) 398:759–71. doi: 10.1016/S0140-6736(21)01234-4 34454674

[68] Maio M Scherpereel A Calabrò L Aerts J Perez SC Bearz A . Tremelimumab as second-line or third-line treatment in relapsed Malignant mesothelioma (DETERMINE): a multicentre, international, randomised, double-blind, placebo-controlled phase 2b trial. Lancet Oncol (2017) 18:1261–73. doi: 10.1016/S1470-2045(17)30446-1 28729154

[69] Reck M Ciuleanu TE Cobo M Schenker M Zurawski B Menezes J . First-line nivolumab plus ipilimumab with two cycles of chemotherapy versus chemotherapy alone (four cycles) in advanced non-small-cell lung cancer: CheckMate 9LA 2-year update. ESMO Open (2021) 6:100273. doi: 10.1016/j.esmoop.2021.100273 34607285 PMC8493593

[70] Mai HQ Chen QY Chen D Hu C Yang K Wen J . Toripalimab or placebo plus chemotherapy as first-line treatment in advanced nasopharyngeal carcinoma: a multicenter randomized phase 3 trial. Nat Med (2021) 27:1536–43. doi: 10.1038/s41591-021-01444-0 34341578

[71] Yang Y Wang Z Fang J Yu Q Han B Cang S . Efficacy and Safety of Sintilimab Plus Pemetrexed and Platinum as First-Line Treatment for Locally Advanced or Metastatic Nonsquamous NSCLC: a Randomized, Double-Blind, Phase 3 Study (Oncology pRogram by InnovENT anti-PD-1-11). J Thorac Oncol (2020) 15:1636–46. doi: 10.1016/j.jtho.2020.07.014 32781263

[72] Zhou C Wu L Fan Y Wang Z Liu L Chen G . Sintilimab plus platinum and gemcitabine as first-line treatment for advanced or metastatic squamous NSCLC: results from a randomized, double-blind, phase 3 trial (ORIENT-12). J Thorac Oncol (2021) 16:1501–11. doi: 10.1016/j.jtho.2021.04.011 34048947

[73] Luo H Lu J Bai Y Mao T Wang J Fan Q . Effect of camrelizumab vs placebo added to chemotherapy on survival and progression-free survival in patients with advanced or metastatic esophageal squamous cell carcinoma: the ESCORT-1st randomized clinical trial. JAMA (2021) 326:916–25. doi: 10.1001/jama.2021.12836 PMC8441593 34519801

[74] Ribas A Puzanov I Dummer R Schadendorf D Hamid O Robert C . Pembrolizumab versus investigator-choice chemotherapy for ipilimumab-refractory melanoma (KEYNOTE-002): a randomised, controlled, phase 2 trial. Lancet Oncol (2015) 16:908–18. doi: 10.1016/S1470-2045(15)00083-2 PMC9004487 26115796

[75] Hamid O Puzanov I Dummer R Schachter J Daud A Schadendorf D . Final analysis of a randomised trial comparing pembrolizumab versus investigator-choice chemotherapy for ipilimumab-refractory advanced melanoma. Eur J Cancer (2017) 86:37–45. doi: 10.1016/j.ejca.2017.07.022 28961465

[76] Fehrenbacher L Spira A Ballinger M Kowanetz M Vansteenkiste J Mazieres J . Atezolizumab versus docetaxel for patients with previously treated non-small-cell lung cancer (POPLAR): a multicentre, open-label, phase 2 randomised controlled trial. Lancet (2016) 387:1837–46. doi: 10.1016/S0140-6736(16)00587-0 26970723

[77] Mazieres J Rittmeyer A Gadgeel S Hida T Gandara DR Cortinovis DL . Atezolizumab versus docetaxel in pretreated patients with NSCLC: final results from the randomized phase 2 POPLAR and phase 3 OAK clinical trials. J Thorac Oncol (2020) 16:140–50. doi: 10.1016/j.jtho.2020.09.022 33166718

[78] Herbst RS Garon EB Kim DW Cho BC Gervais R Perez-Gracia JL . Five year survival update from KEYNOTE-010: pembrolizumab versus docetaxel for previously treated, programmed death-ligand 1-positive advanced NSCLC. J Thorac Oncol (2021) 16:1718–32. doi: 10.1016/j.jtho.2021.05.001 34048946

[79] Belin L Tan A De Rycke Y Dechartres A . Progression-free survival as a surrogate for overall survival in oncology trials: a methodological systematic review. Br J Cancer (2020) 122:1707–14. doi: 10.1038/s41416-020-0805-y PMC7250908 32214230

[80] Tan A Porcher R Crequit P Ravaud P Dechartres A . Differences in treatment effect size between overall survival and progression-free survival in immunotherapy trials: A meta-epidemiologic study of trials with results posted at clinicalTrials.gov. J Clin Oncol (2017) 35:1686–94. doi: 10.1200/JCO.2016.71.2109 28375786

[81] Uno H Claggett B Tian L Inoue E Gallo P Miyata T . Moving beyond the hazard ratio in quantifying the between-group difference in survival analysis. J Clin Oncol (2014) 32:2380–5. doi: 10.1200/JCO.2014.55.2208 PMC4105489 24982461

[82] Fleming TR Rothmann MD Lu HL . Issues in using progression-free survival when evaluating oncology products. J Clin Oncol (2009) 27:2874–80. doi: 10.1200/JCO.2008.20.4107 PMC2698020 19414672

[83] Seymour L Bogaerts J Perrone A Ford R Schwartz LH Mandrekar S . iRECIST: guidelines for response criteria for use in trials testing immunotherapeutics. Lancet Oncol (2017) 18:e143–52. doi: 10.1016/S1470-2045(17)30074-8 PMC5648544 28271869

[84] Burzykowski T Molenberghs G Buyse M . Validation of surrogate end points in multiple randomized clinical trials with failure time end points. J R Stat Soc Ser C (Applied Stat) (2001) 50:405–22. doi: 10.1111/1467-9876.00244

[85] U.S. Food and drug administration: clinical trial endpoints for the approval of cancer drugs and biologics: guidance for industry. US Food Drug Adm (2018). Available at: https://www.fda.gov/regulatory-information/search-fda-guidance-documents/clinical-trial-endpoints-approval-cancer-drugs-and-biologics.

