# Supplementary material for: Surrogate endpoints for overall survival in randomized clinical trials testing immune checkpoint inhibitors: a systematic review and meta-analysis
Source: Front Immunol. 2024 Jan 29;15:1340979. doi: 10.3389/fimmu.2024.1340979 (PMC10859450; doi:10.3389/fimmu.2024.1340979)
Supplement: Supplementary file 1 [file DataSheet_1.docx]

**Supplementary Appendix**

[**ABBREVIATIONS AND ACRONYMS** 2](#_Toc134688618)

[**SUPPLEMENTARY METHODS** 3](#_Toc134688619)

[**SUPPLEMENTARY RESULTS** 6](#_Toc134688620)

[**Figure S1.** Example of how treatment effect values on (m)PFS vary by changing the measure that quantifies the effect 6](#_Toc134688621)

[**Figure S2.** PRISMA flow diagram of study selection process 8](#_Toc134688622)

[**Figure S3.** Correlations between effects of ICI plus chemotherapy on OS and the potential surrogate endpoints, PFS (panel A and C) and mPFS (panel B and D) 9](#_Toc134688624)

[**Figure S4.** Correlations between effects of ICI plus ICI or other treatment(s) on OS and the potential surrogate endpoints, PFS (panel A and C) and mPFS (panel B and D) 10](#_Toc134688625)

[**Table S1.** Characteristics of RCTs included in the analysis 11](#_Toc134688626)

[**Table S2.** Quality assessment of trials according to the Cochrane Risk of bias tool 15](#_Toc134688627)

[**Table S3.** Treatment effect estimates and their ratios 17](#_Toc134688628)

[**Table S4.** Pairwise agreement between statistical significance of HR_OS_ and each surrogate measure 20](#_Toc134688629)

[**REFERENCES** 21](#_Toc134688630)

## **ABBREVIATIONS AND ACRONYMS**

CI = confidence interval

HR = hazard ratio

ICI = immune checkpoint inhibitor

IPD = individual patient-level data

KM = Kaplan-Meier

ln-HR = natural logarithm of hazard ratio

ln-rRMST = natural logarithm of ratio of restricted mean survival time

mPFS = modified progression-free survival

OS = overall survival

PFS = progression-free survival

PH = proportional hazards

R2 = coefficient of determination

RCT = randomized clinical trial

RMST = restricted mean survival time

rRMST = ratio of restricted mean survival time

SE = standard error

STE = surrogate threshold effect

WLS = weighted linear regression

## **SUPPLEMENTARY METHODS**

**Statistical analysis**

The data extracted from the included three-arm trials were treated as two separate comparisons, with the control arm being duplicated in both comparisons. For this reason, our unit of analysis was the comparison between pairs of treatment arms, and not the trial. Each pairwise comparison was categorized according to the type of treatment administered in the experimental arm: ICI alone, ICI plus chemotherapy, or ICI plus ICI or other treatment(s).

*Estimation of treatment effect size for OS, PFS, and mPFS*

For each comparison, we analyzed the reconstructed pseudo individual patient-level data (IPD). We used Cox proportional hazard (PH) regression models to estimate hazard ratio for overall survival (HR_OS_) and for progression-free survival (HR_PFS_), their 95% confidence intervals (95% CI), and the standard error (SE) of the natural logarithm of the HRs (ln-HR). A HR less than one favored the experimental treatment.

For both OS and PFS, we tested for non-PHs using the Grambsch-Therneau test.^1^

The Kaplan-Meier (KM) estimate of the PFS function was used to calculate the restricted mean survival time (RMST; i.e., the area under the survival function for a specified time horizon [0 ‐ *t^*^*]), which is the mean survival time of all subjects in the study population followed up to *t^*^*.^2^ We calculated the RMST_PFS_ in the control and experimental group prespecifying the time horizon *t^*^* as the minimum of the largest observed event time in each of the two groups.

We calculated rRMST_PFS_ as the ratio of RMST_PFS_ in the control group to RMST_PFS_ in the experimental group to obtain a measure in the same direction of the HR (i.e., a rRMST_PFS_ lower than one indicated a protective effect of the experimental treatment). The corresponding 95% CI and the SE of ln-rRMST_PFS_ were also estimated.

For modified PFS (mPFS), we calculated the ln-HR_mPFS_ and ln-rRMST_mPFS_, and their SEs, over the 1000 simulated dataset of matched PFS-OS pseudo IPD, taking their averages as the estimates for each treatment comparison. mPFS was calculated omitting the events of disease progression (but not deaths) within 3 months after randomization.

**Supplementary Figure S1** provides an example of how treatment effect values on (m)PFS vary by changing the measure that quantifies the effect.

*Estimation of differences in treatment effect size between OS-PFS and OS-mPFS within each pairwise treatment comparison*

Within each treatment comparison, we compared differences in treatment effect measures by using the ratio of the surrogate effect measure (*s*; i.e., HR_PFS_, HR_mPFS_, rRMST_PFS_, or rRMST_mPFS_) to the final effect measure (*f*; i.e., HR_OS_). A ratio $\frac{s}{f}<1$ indicated that the treatment effect size on the surrogate measure overestimates the treatment effect size on the final measure. Since surrogate and final estimates were obtained from the same pairwise treatment comparison, the ratio $\frac{s}{f}$ was not independent. To take into account this dependency, the SE of the natural logarithm of the ratio was calculated as:

$$SE\left( \ln\left( \frac{s}{f} \right) \right)=\sqrt{Var(ln(s))+Var(ln(f))-\rho\times2\times SE(ln(s))\times SE(ln(f))}$$

where *ρ* is the within-study correlation between the two effects.

A bootstrapping method was used to estimate the within-study correlations between the treatment effects on surrogate and final measures, by drawing 100 bootstrap samples with replacement from a subset of 100 matched PFS-OS pseudo IPD generated with the same algorithm used to derive mPFS. For each bootstrap sample, the treatment effects on surrogate and final outcomes were estimated by fitting Cox PH regression and RMST analysis, as described above. The Pearson correlation coefficient between the two outcomes was then obtained. As a measure of within-study correlation $\rho$ for the two pairs of effects, we used the average Pearson correlation coefficient calculated over the 100 simulated dataset of matched PFS-OS pseudo IPD.

To evaluate whether using PFS instead of OS in the included comparisons would have changed drug approval decisions, we assessed the agreement between HR_OS_ and HR_PFS_ in terms of statistical significance (i.e., p-value <0.05) using Kohen’s Kappa coefficient and p-value of the McNemar’s test.

*Pooled treatment effect sizes and pooled differences of treatment effect sizes*

We combined surrogate and final treatment effect sizes for each pairwise treatment comparison using a random effects meta-analysis. The heterogeneity across trials was assessed with the Cochrane Q test and the I^2^ statistic (the proportion of total variation contributed by between-trial variance).^3^

Similarly, we combined across trials the ratios of the surrogate effect measure to the final effect measure.

*Surrogate endpoint validation*

We used a correlation approach to assess the surrogacy value of each potential surrogate endpoint as previously described.^4^ A weighted linear regression (WLS) model was used to quantify the association between the treatment effect on the final endpoint (HR_OS_) and each surrogate measure (HR_PFS_, HR_mPFS_, rRMST_PFS_, and rRMST_mPFS_) in strata of treatment type administered in the experimental arms (i.e., ICI alone, ICI plus chemotherapy, ICI plus ICI or other treatment(s)). The model was weighted by the number of subjects randomized in each pairwise treatment comparison. In the model, treatment effects were fitted on a log scale.

The coefficient of determination (R^2^) estimated from the model was used to measure the proportion of variance in treatment effect on OS that is predictable from the treatment effect on the surrogate, and therefore to quantify the surrogacy value at trial-level of each potential surrogate endpoint. The 95% CI for R^2^ was estimated by bootstrap analysis with 1000 samples. According to ReSEEM guidelines^5^, R^2^ values equal to or higher than 0.7 represent *strong* correlations (and was therefore suggestive of surrogacy), values between 0.69 and 0.5 represent *moderate* correlations, and values lower than 0.5 represent *weak* correlations. The slope of the regression line was also reported as an alternative measure of surrogacy. For the treatment effects to be associated, we required that the slope significantly differed from zero.

Finally, we calculated the surrogate threshold effect (STE), defined as the minimum treatment effect on the surrogate endpoint necessary to predict a significant OS benefit in a future trial. Graphically, STE is the intersection of the upper limit of the 95% prediction band and the horizontal line representing the predicted HR_OS_ equal to 1 (null effect).^6^ The 95% prediction band was calculated from the weighted regression model used to derive the coefficient of determination R^2^, and it was based on the weight assigned to the HR being predicted for a future trial. In the calculation of the prediction band, we considered a future trial with an expected sample size equal to the average number of patients observed in the included trials.

All analyses were performed with SAS software v. 9.4 (SAS Institute, Cary, North Carolina, USA) and R software (version 3.6.0).

## **SUPPLEMENTARY RESULTS**

### **Figure S1.** Example of how treatment effect values on (m)PFS vary by changing the measure that quantifies the effect


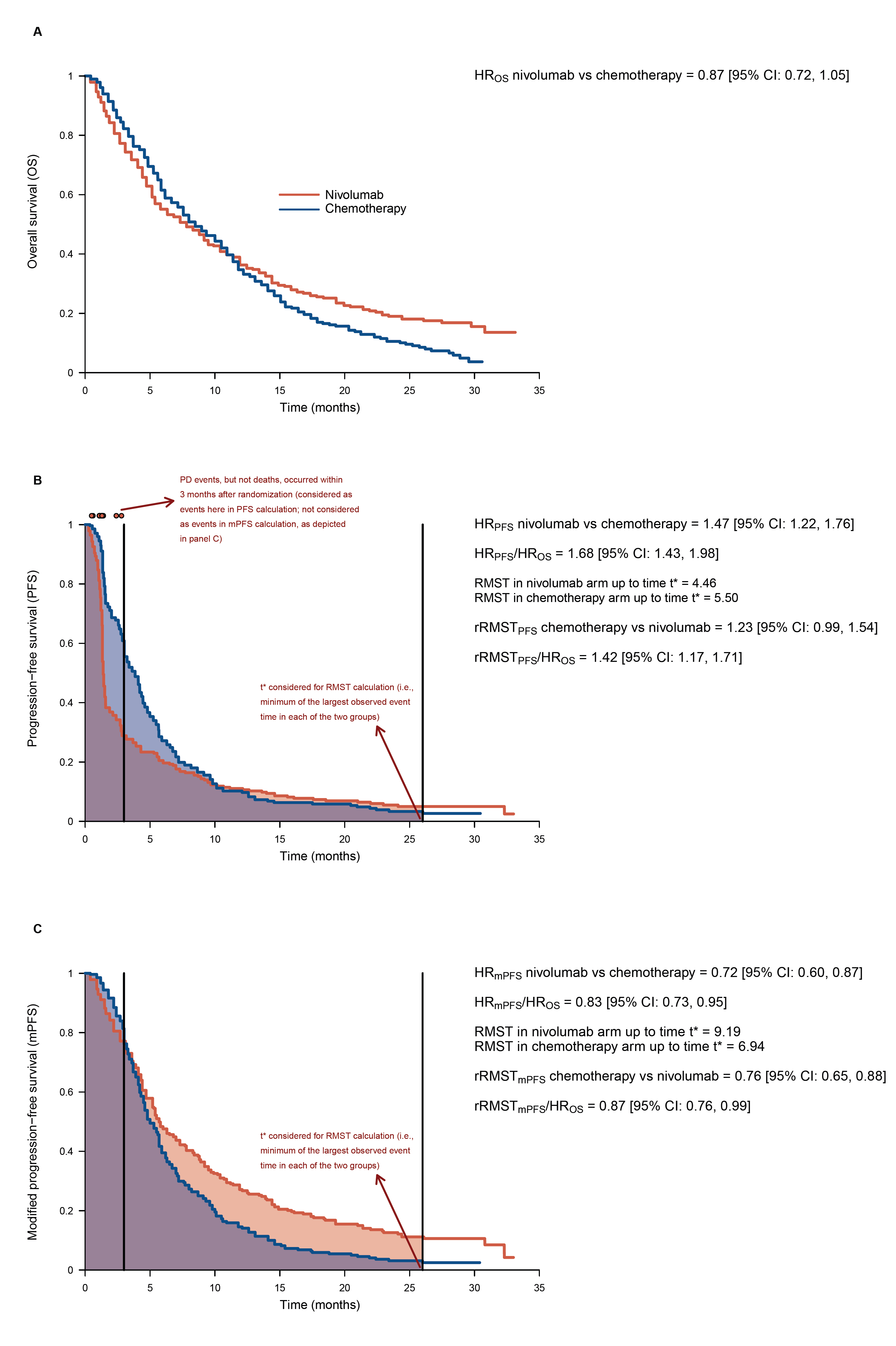


**Figure S1 legend.** The figure shows the KM curves for OS and PFS derived from the pseudo IPD extracted from the published curves of an included phase III randomized clinical trial (RCT; CheckMate 331^8^), comparing nivolumab versus standard chemotherapy in patients with relapsed small-cell lung cancer. The figure also shows the mPFS curves calculated from one of the 1000 matched PFS-OS pseudo IPD dataset.

Along with the KM curves, the computed treatment effect sizes and differences of treatment effect sizes were also reported.

It is to be noted that the ratio of RMST in the experimental to the RMST in the control group is usually computed to quantify the relative treatment effect (i.e., a ratio greater than one indicates superiority of the experimental treatment). However, in this study, we calculated rRMST_PFS_ as the ratio of RMST_PFS_ in the control group to RMST_PFS_ in the experimental group to obtain a measure in the same direction of the HR. Therefore, a rRMST_PFS_ lower than one indicates a protective effect of the experimental treatment.

It has been suggested that the rRMST is a more robust and comprehensible summary measure of treatment effect than the HR, particularly when the PH assumption is violated.^9^ For instance, if *t^*^* is 24 months, the RMST_PFS_ measures the average number of months survived free of progression over 2 years. When rRMST_PFS_ is <1, (1 - rRMST_PFS_) measures the relative loss in 2-year life expectancy associated with the control treatment versus the experimental. When rRMST_PFS_ is >1, (rRMST_PFS_ - 1) measures the relative gain in 2-year life expectancy associated with the control treatment versus the experimental.

**Panel A** shows the KM OS curves, and the HR_OS_ estimated from the Cox PH regression model (HR_OS_=0.87, indicating that the hazard of death was 13% lower in the nivolumab arm than in the chemotherapy arm).

**Panel B** shows the KM PFS curves, and the HR_PFS_ estimated from the Cox PH regression model (HR_PFS_=1.47, indicating that the hazard of progression was 47% greater in the nivolumab arm than in the chemotherapy arm), the ratio of the HR_PFS_ to HR_OS_ (HR_PFS_/HR_OS_=1.68, indicating that the HR_PFS_ underestimated the treatment effect observed for OS), the restricted mean progression-free survival up to *t^*^*=26 months (that is the area under the KM PFS curve up to *t^*^*, where *t^*^* is the minimum of the largest observed event time in each of the two group) for chemotherapy (RMST_PFS-chemotherapy_=5.5, represented by the blue area, estimating the average number of months survived progression-free over 26 months in the chemotherapy arm) and nivolumab group (RMST_PFS-nivolumab_=4.5, represented by the red area, estimating the average number of months survived progression-free over 26 months in the nivolumab arm); the ratio of the two RMST areas (rRMST_PFS_=1.23, indicating a relative gain of 23% in 26-month life expectancy associated with the control treatment versus the experimental); the ratio of the rRMST_PFS_ to HR_OS_, equal to 1.42, indicating that the rRMST_PFS_ underestimated the treatment effect observed for OS.

**Panel C** shows the KM mPFS curves, and the HR_mPFS_ estimated from the Cox PH regression model (HR_mPFS_=0.72, indicating that the hazard of modified progression was 28% lower in the nivolumab arm than in the chemotherapy arm), the ratio of the HR_mPFS_ to HR_OS_ (HR_mPFS_/HR_OS_=0.83, indicating that the HR_PFS_ overestimated the treatment effect observed for OS), the restricted mean progression-free survival up to *t^*^*=26 months for chemotherapy (RMST_mPFS-chemotherapy_=6.9, represented by the blue area) and nivolumab group (RMST_mPFS-nivolumab_=9.2, represented by the red area); the ratio of the two RMST areas (rRMST_mPFS_=0.76, indicating a relative loss of 24% in 26-month life expectancy associated with the control treatment versus the experimental); the ratio of the rRMST_mPFS_ to HR_OS_, equal to 0.87, indicating that the rRMST_mPFS_ overestimated the treatment effect observed for OS.

### **Figure S2.** PRISMA flow diagram of study selection process


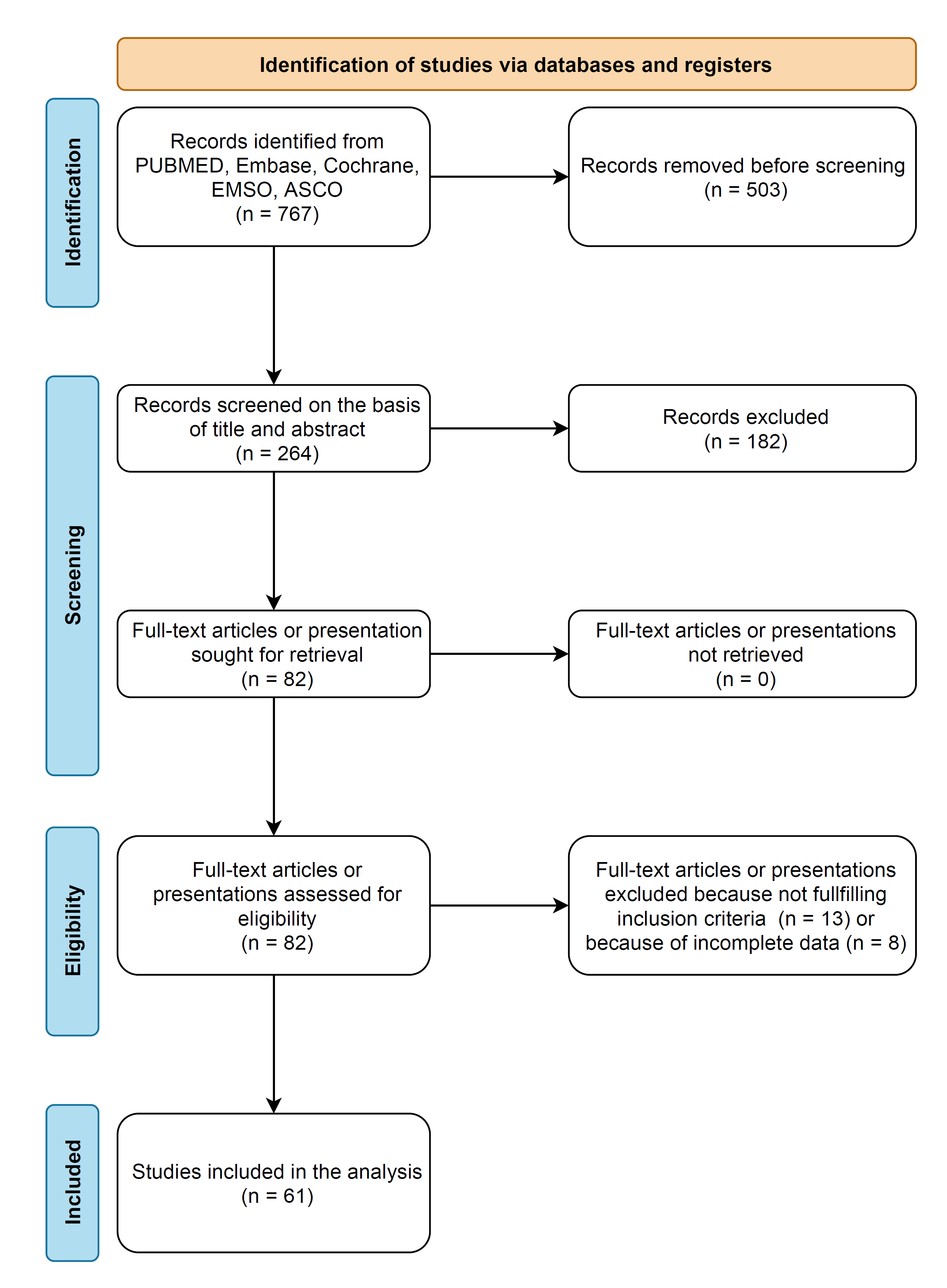


**Search terms and filters**

Search terms: “CTLA-4”, “cytotoxic T-lymphocyte-associated protein 4”, “PD-1”, “programmed death receptor 1”, “PD-L1”, “immune checkpoint inhibitor”, “ipilimumab”, “tremelimumab”, “nivolumab”, “pembrolizumab”, “durvalumab”, “atezolizumab”, “cemiplimab”, “spartalizumab”, “avelumab”, “toripalimab”, “dostarlimab”, “balstilimab”, “penpulimab”, “retifanlimab”, “sintilimab”.

Filter for RCTs - Cochrane Highly Sensitive Search Strategy for identifying randomized trials in MEDLINE: sensitivity- and precision-maximizing version (2008 revision; https://training.cochrane.org/handbook/version-6/chapter-4-tech-suppl)

(randomized controlled trial [pt] OR controlled clinical trial [pt] OR randomized [tiab] OR placebo [tiab] OR drug therapy [sh] OR randomly [tiab] OR trial [tiab] OR groups [tiab]) NOT (animals [mh] NOT humans [mh])

**Figure S3.** Correlations between effects of ICI plus chemotherapy on OS and the potential surrogate endpoints, PFS (panel A and C) and mPFS (panel B and D)

| 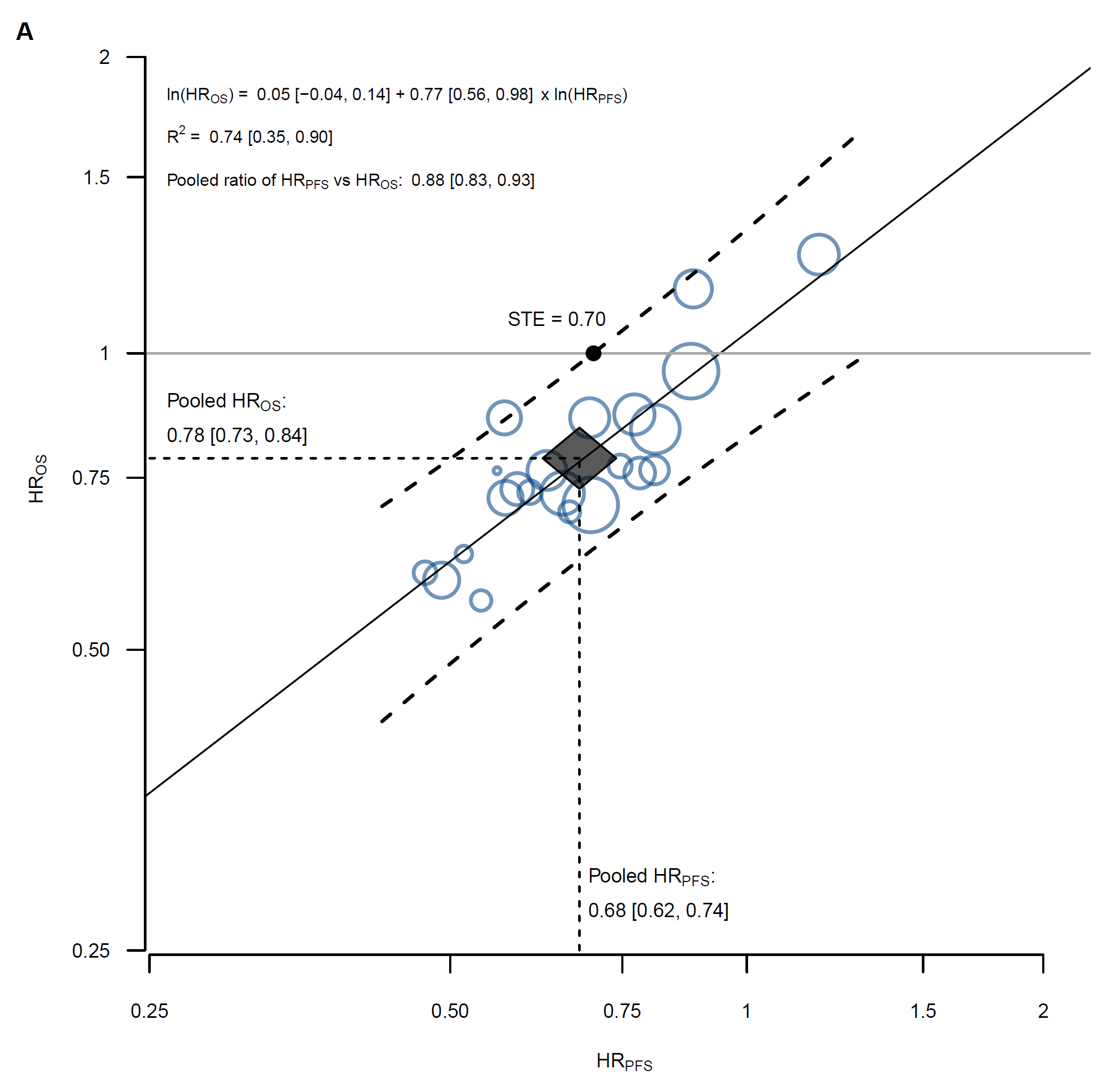 | 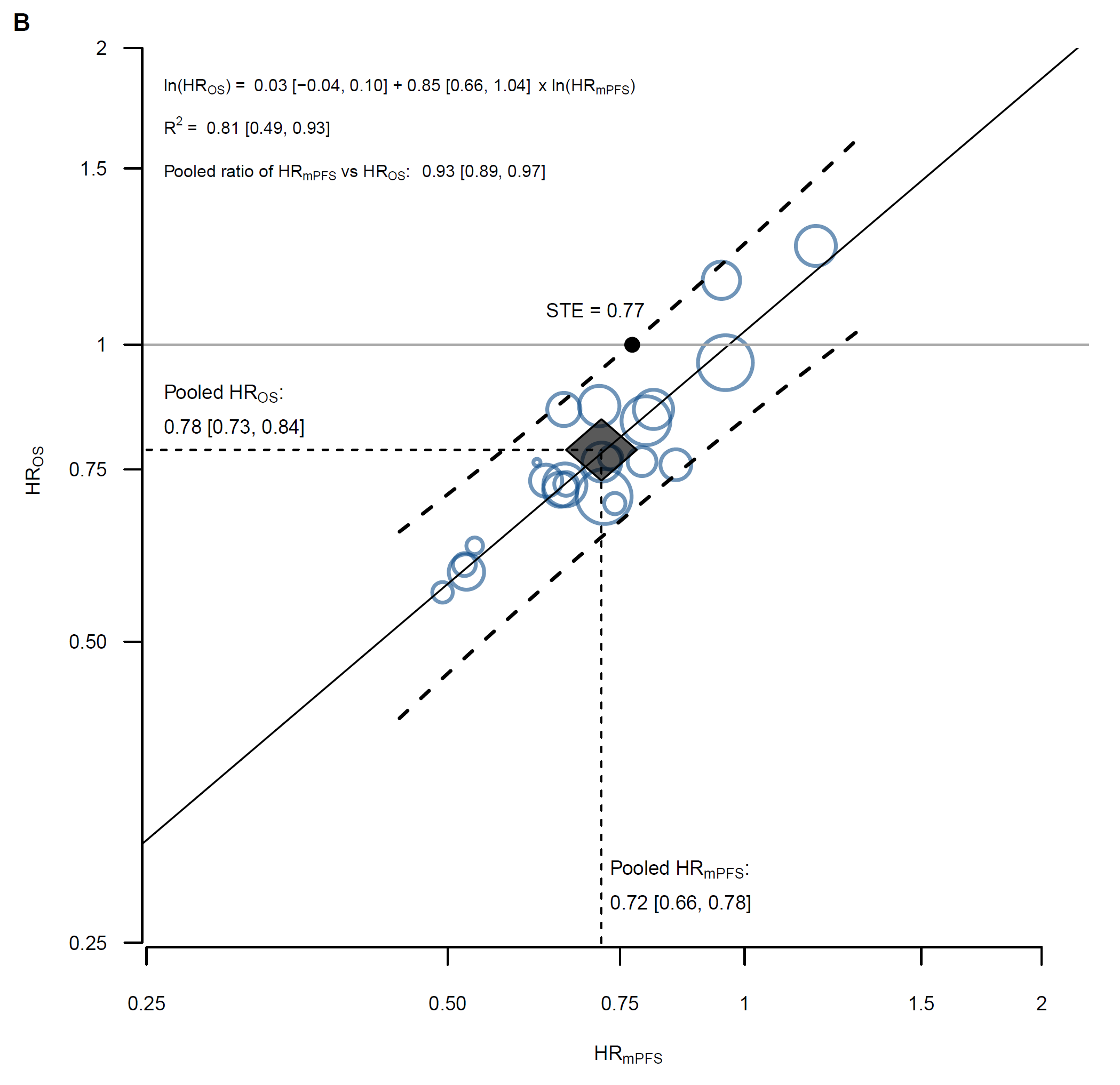 |
| --- | --- |
| 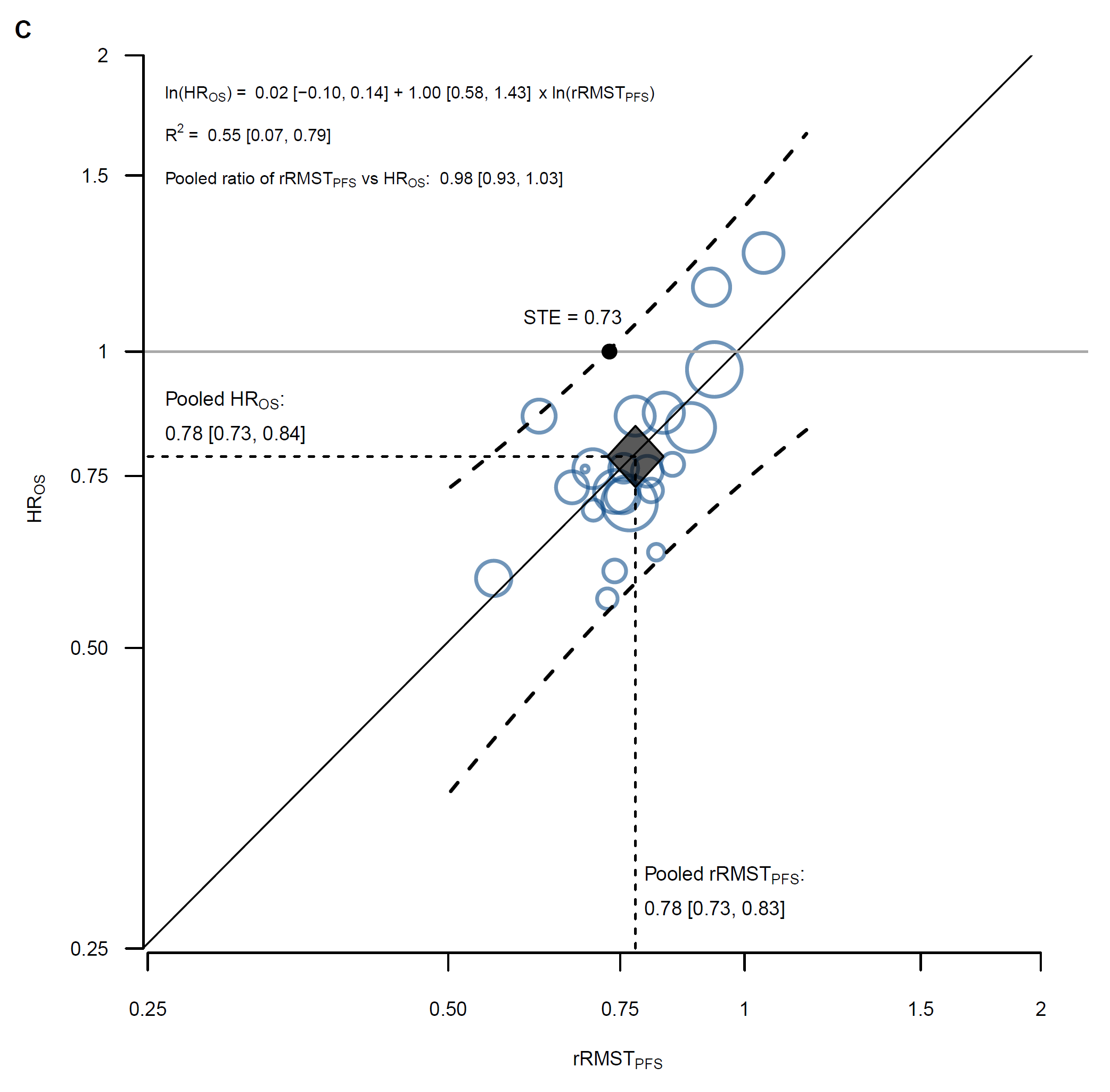 | 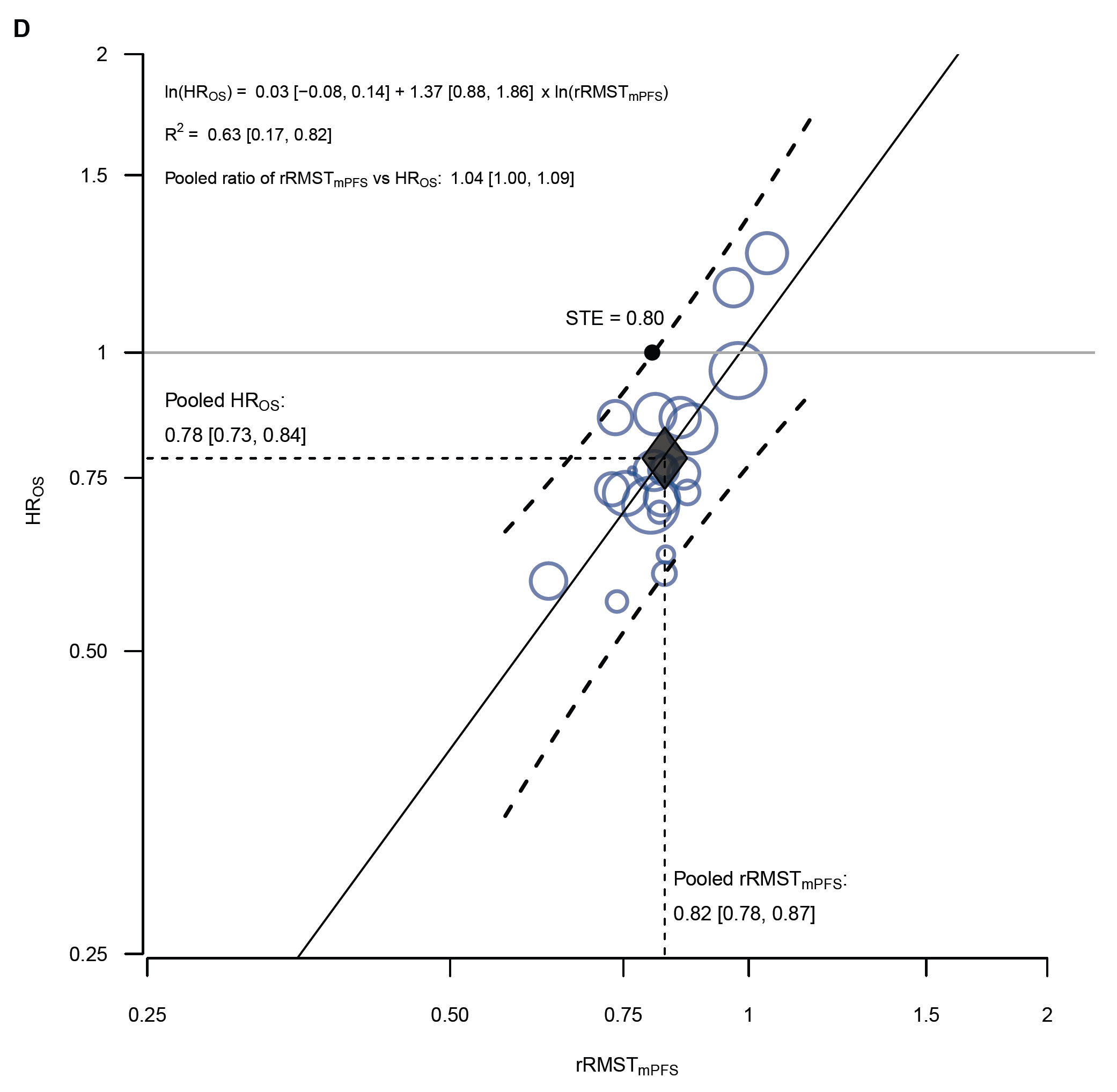 |

**Figure S3 legend.** The figure shows the correlations between effects of ICI plus chemotherapy on OS and the potential surrogate endpoints, PFS (panel A and C) and mPFS (panel B and D). The treatment effects are measured by HR for OS, and by the HR and the rRMST for the two surrogate endpoints.

Each circle represents a comparison, and the surface area of the circle is proportional to the number of patients in the corresponding comparison. Straight line represents weighted regression line. Dashed lines represent 95% prediction bands based on the values predicted by the weighted regression model. The surrogate threshold effect (STE) is represented by the intersection point between the horizonal line y=1 and the upper 95% prediction band.

Black diamond indicates the meta-analytic pooled estimate. The diamond’s width represents the 95% CI of the surrogate pooled estimate, and height represents the 95% CI of the HR_OS_ pooled estimate.

The surrogacy equation between the log-transformed treatment effects and the ln-HR_OS_ estimated from the weighted linear regression, the R^2^ coefficient, and the pooled ratio between surrogate endpoint and HR_OS_ were also reported with their 95% CI (displayed in square brackets).

### **Figure S4.** Correlations between effects of ICI plus ICI or other treatment(s) on OS and the potential surrogate endpoints, PFS (panel A and C) and mPFS (panel B and D)

| 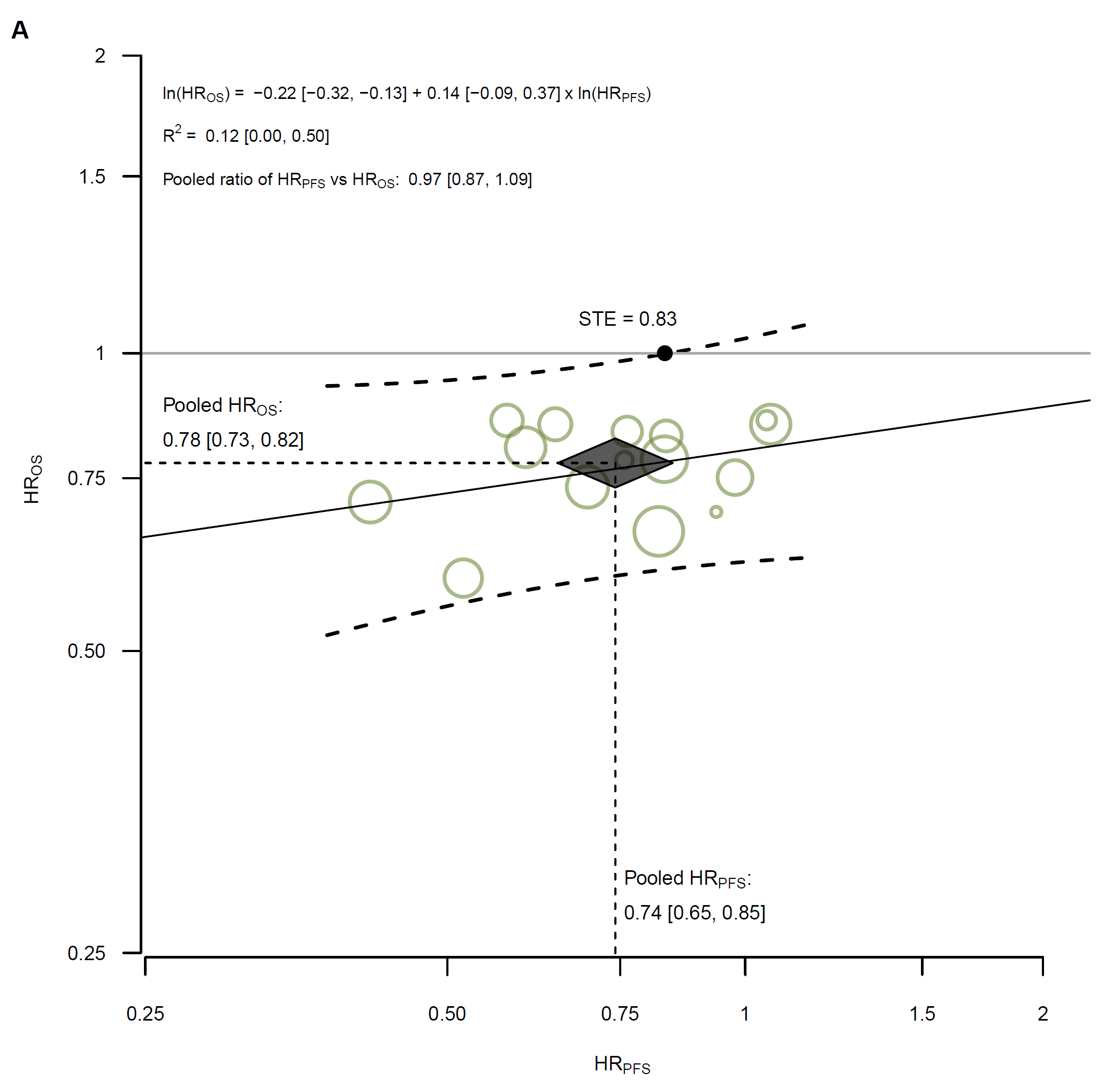 | 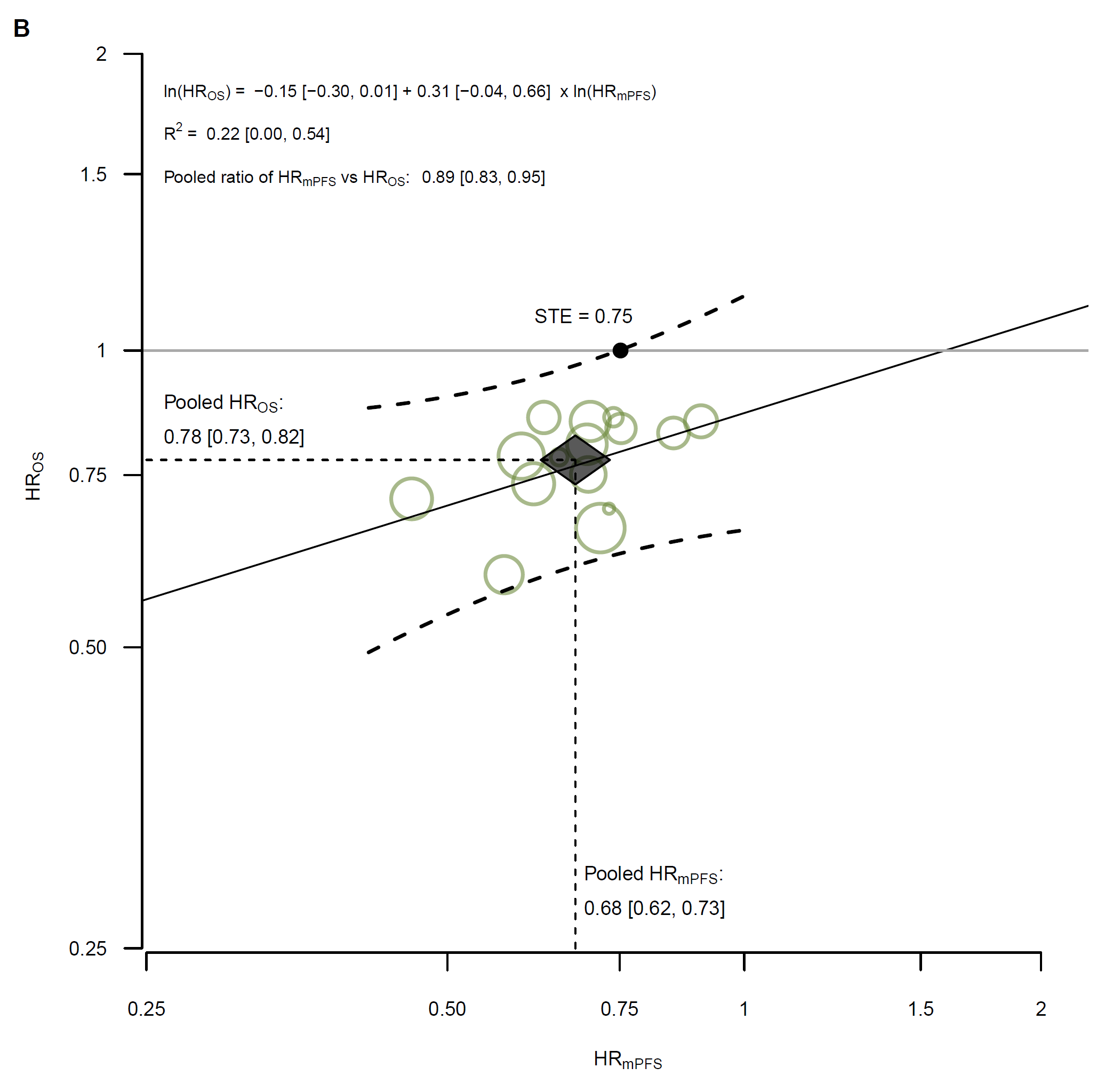 |
| --- | --- |
| 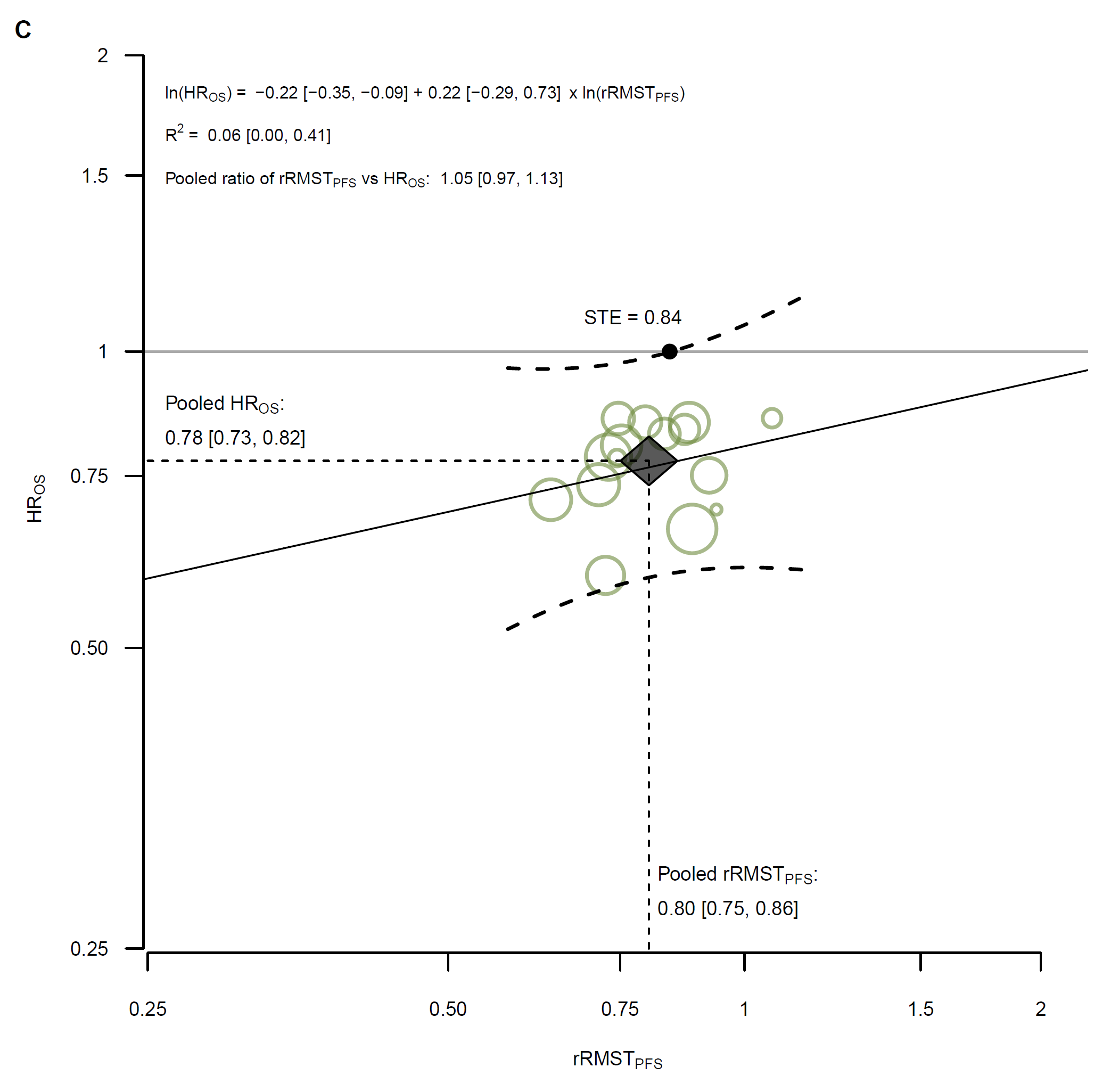 | 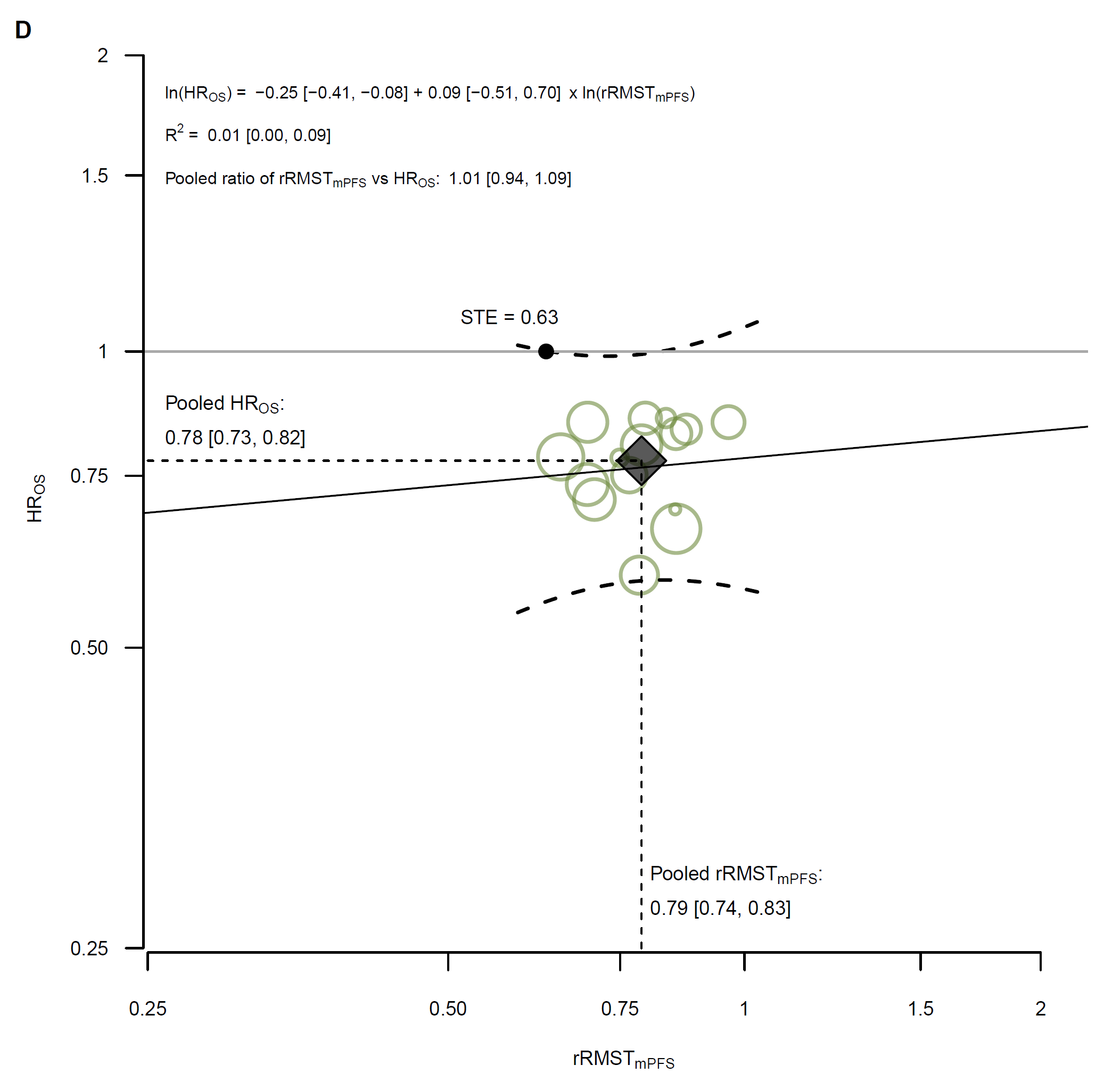 |

**Figure S4 legend.** The figure shows the correlations between effects of ICI plus ICI or other treatment(s) on OS and the potential surrogate endpoints, PFS (panel A and C) and mPFS (panel B and D). The treatment effects are measured by HR for OS, and by the HR and the rRMST for the two surrogate endpoints.

Each circle represents a comparison, and the surface area of the circle is proportional to the number of patients in the corresponding comparison. Straight line represents weighted regression line. Dashed lines represent 95% prediction bands based on the values predicted by the weighted regression model. The surrogate threshold effect (STE) is represented by the intersection point between the horizonal line y=1 and the upper 95% prediction band.

Black diamond indicates the meta-analytic pooled estimate. The diamond’s width represents the 95% CI of the surrogate pooled estimate, and height represents the 95% CI of the HR_OS_ pooled estimate.

The surrogacy equation between the log-transformed treatment effects and the ln-HR_OS_ estimated from the weighted linear regression, the R^2^ coefficient, and the pooled ratio between surrogate endpoint and HR_OS_ were also reported with their 95% CI (displayed in square brackets).

### **Table S1.** Characteristics of RCTs included in the analysis

| **Trial** | **NCT** | **Publication** | **Tumor site** | **Phase** | **Line** | **Primary endpoint** | **Treatment type (experimental vs control)** | **Arm (experimental vs control)** | **N (experimental \ control)** | **PH assumption was not rejected for ^a^** |
| --- | --- | --- | --- | --- | --- | --- | --- | --- | --- | --- |
|  | NCT00324155 | Robert C et al. 2011^10^ | Melanoma | III | I | OS | ICI + CT vs CT | Ipilimumab + Dacarbazine vs Dacarbazine | 250 \ 252 | Both OS and PFS |
|  | NCT01450761 | Reck M et al. 2016^11^ | Small Cell Lung Cancer | III | I | OS | ICI + CT vs CT | Ipilimumab + Platinum-Etoposide vs Placebo + Platinum-Etoposide | 478 \ 476 | Both OS and PFS |
| CheckMate 017 | NCT01642004 | Borghaei H et al. 2021^12^ | Non-Small Cell Lung Cancer | III | >I | OS | ICI vs CT | Nivolumab vs Docetaxel | 135 \ 137 | OS or PFS only |
| CheckMate 025 | NCT01668784 | Motzer R et al. 2015^13^ | Renal Cancer | III | >I | OS | ICI vs targeted therapy | Nivolumab vs Everolimus | 410 \ 411 | Both OS and PFS |
| CheckMate 057 | NCT01673867 | Borghaei H et al. 2021^12^ | Non-Small Cell Lung Cancer | III | >I | OS | ICI vs CT | Nivolumab vs Docetaxel | 292 \ 290 | None |
| KEYNOTE-002 | NCT01704287 | Ribas A et al. 2015^14^ and Hamid O et al. 2017^15^ | Melanoma | II | >I | OS+PFS | ICI vs CT | Pembrolizumab 2 mg/kg vs Investigator's Choice of Chemotherapy | 180 \ 179 | Both |
| KEYNOTE-002 | NCT01704287 | Ribas A et al. 2015^14^ and Hamid O et al. 2017^15^ | Melanoma | II | >I | OS+PFS | ICI vs CT | Pembrolizumab 10 mg/kg vs Investigator's Choice of Chemotherapy | 181 \ 179 | Both |
| CheckMate 037 | NCT01721746 | Larkin J et al. 2018^16^ | Melanoma | III | >I | OS+ORR | ICI vs CT | Nivolumab vs Investigator's Choice of Chemotherapy | 272 \ 133 | OS or PFS only |
| CheckMate 066 | NCT01721772 | Robert C et al. 2015^17^ | Melanoma | III | I | OS | ICI vs CT | Nivolumab vs Dacarbazine | 210 \ 208 | None |
| DETERMINE | NCT01843374 | Maio M et al. 2017^18^ | Mesothelioma | II | >I | OS | ICI vs placebo | Tremelimumab vs Placebo | 382 \ 189 | OS or PFS only |
| POPLAR | NCT01903993 | Fehrenbacher L et al. 2016^19^ and Mazieres J et al. 2020^20^ | Non-Small Cell Lung Cancer | II | >I | OS | ICI vs CT | Atezolizumab vs Docetaxel | 144 \ 143 | OS or PFS only |
| KEYNOTE-010 | NCT01905657 | Herbst RS et al. 2021^21^ | Non-Small Cell Lung Cancer | II/III | >I | OS+PFS | ICI vs CT | Pembrolizumab vs Docetaxel | 690 \ 343 | OS or PFS only |
| OAK | NCT02008227 | Fehrenbacher L et al. 2018^22^ and Mazieres J et al. 2020^20^ | Non-Small Cell Lung Cancer | III | >I | OS | ICI vs CT | Atezolizumab vs Docetaxel | 613 \ 612 | OS or PFS only |
| KEYNOTE-021 | NCT02039674 | Awad MM et al. 2020^23^ | Non-Small Cell Lung Cancer | I/II | I | ORR | ICI + CT vs CT | Pembrolizumab + Pemetrexed + Carboplatin vs Pemetrexed + Carboplatin | 60 \ 63 | Both OS and PFS |
| CheckMate 026 | NCT02041533 | Carbone DP et al. 2017^24^ | Non-Small Cell Lung Cancer | III | I | PFS | ICI vs CT | Nivolumab vs Investigator's Choice of Chemotherapy | 211 \ 212 | OS or PFS only |
| CheckMate 141 | NCT02105636 | Ferris RL et al. 2016^25^ | Head and Neck Cancer | III | >I | OS | ICI vs CT | Nivolumab vs Investigator's Choice of Chemotherapy | 240 \ 121 | None |
| KEYNOTE-024 | NCT02142738 | Reck M et al. 2016^26^ and Reck M et al. 2019^27^ | Non-Small Cell Lung Cancer | III | I | PFS | ICI vs CT | Pembrolizumab vs Investigator's Choice of Platinum-based Chemotherapy | 154 \ 151 | OS or PFS only |
| KEYNOTE-042 | NCT02220894 | Mok TSK et al. 2019^28^ | Non-Small Cell Lung Cancer | III | I | OS | ICI vs CT | Pembrolizumab vs Carboplatin + Paclitaxel/Pemetrexed | 637 \ 637 | None |
| CheckMate 214 | NCT02231749 | Motzer R et al. 2018^29^ | Renal Cancer | III | I | OS+PFS | ICI + ICI vs anti-angiogenesis agent | Nivolumab + Ipililumab vs Sunitinib | 425 \ 422 | Both OS and PFS |
| KEYNOTE-040 | NCT02252042 | Cohen EEW et al. 2019^30^ | Head and Neck Cancer | III | >I | OS | ICI vs CT | Pembrolizumab vs Investigator's Choice of Chemotherapy | 247 \ 248 | None |
| KEYNOTE-045 | NCT02256436 | Bellmunt J et al. 2017^31^ | Urothelial Cancer | III | >I | OS+PFS | ICI vs CT | Pembrolizumab vs Investigator's Choice of Chemotherapy | 270 \ 272 | None |
| ATTRACTION-2 | NCT02267343 | Chen LT et al. 2020^32^ | Gastric Cancer | III | >I | OS | ICI vs placebo | Nivolumab vs Placebo | 330 \ 163 | Both OS and PFS |
| ARCTIC | NCT02352948 | Planchard D et al. 2020^33^ | Non-Small Cell Lung Cancer | III | >I | OS+PFS | ICI + ICI vs CT | Durvalumab + Tremelimumab vs SoC | 174 \ 118 | Both OS and PFS |
| ARCTIC | NCT02352948 | Planchard D et al. 2020^33^ | Non-Small Cell Lung Cancer | III | >I | OS+PFS | ICI vs CT | Durvalumab vs SoC | 62 \ 64 | Both OS and PFS |
| IMpower150 | NCT02366143 | Socinski MA et al. 2018^34^ and Socinski MA et al. 2021^35^ | Non-Small Cell Lung Cancer | III | I | OS+PFS | ICI + CT + anti-angiogenesis agent vs anti-angiogenesis agent + CT | Atezolizumab + Bevacizumab + Carboplatin + Paclitaxel vs Bevacizumab + Carboplatin + Paclitaxel | 356 \ 336 | Both OS and PFS |
| IMpower130 | NCT02367781 | West H et al. 2019^36^ | Non-Small Cell Lung Cancer | III | I | OS+PFS | ICI + CT vs CT | Atezolizumab + Carboplatin + Nab-Paclitaxel vs Carboplatin + Nab-Paclitaxel | 451 \ 228 | Both OS and PFS |
| IMpower131 | NCT02367794 | Jotte R et al. 2020^37^ | Non-Small Cell Lung Cancer | III | I | OS+PFS | ICI + CT vs CT | Atezolizumab + Carboplatin + Nab-Paclitaxel vs Carboplatin + Nab-Paclitaxel | 343 \ 340 | OS or PFS only |
| JAVELIN Lung 200 | NCT02395172 | Barlesi F et al. 2018^38^ and Park K et al. 2021^39^ | Non-Small Cell Lung Cancer | III | >I | OS | ICI vs CT | Avelumab vs Docetaxel | 264 \ 265 | OS or PFS only |
| IMpower110 | NCT02409342 | Herbst RS et al. 2020^40^ | Non-Small Cell Lung Cancer | III | I | OS | ICI vs CT | Atezolizumab vs Investigator's Choice of Chemotherapy | 107 \ 98 | OS or PFS only |
| IMpassion130 | NCT02425891 | Emens LA et al. 2021^41^ | Breast Cancer | III | I | OS+PFS | ICI + CT vs CT | Atezolizumab + Nab-Paclitaxel vs Placebo + Nab-Paclitaxel | 185 \ 184 | Both OS and PFS |
| MYSTIC | NCT02453282 | Rizvi NA et al. 2020^42^ | Non-Small Cell Lung Cancer | III | I | OS+PFS | ICI + ICI vs CT | Durvalumab + Tremelimumab vs Investigator's Choice of Platinum-based Chemotherapy | 163 \ 162 | None |
| MYSTIC | NCT02453282 | Rizvi NA et al. 2020^42^ | Non-Small Cell Lung Cancer | III | I | OS+PFS | ICI vs CT | Durvalumab vs Investigator's Choice of Platinum-based Chemotherapy | 163 \ 162 | None |
| CheckMate 227 | NCT02477826 | Hellmann MD et al. 2019^43^ and Paz-Ares L et al. 2021^44^ | Non-Small Cell Lung Cancer | III | I | OS+PFS | ICI + ICI vs CT | Nivolumab + Ipililumab vs Investigator's Choice of Platinum-based Chemotherapy | 396 \ 397 | None |
| CheckMate 331 | NCT02481830 | Spigel DR et al. 2021^8^ | Small Cell Lung Cancer | III | >I | OS | ICI vs CT | Nivolumab vs Topotecan/Amrubicin | 284 \ 285 | None |
| DANUBE | NCT02516241 | Powles T et al. 2020^45^ | Urothelial Cancer | III | I | OS | ICI vs CT | Durvalumab vs Gemcitabine + Cisplatin/Carboplatin | 209 \ 207 | None |
| DANUBE | NCT02516241 | Powles T et al. 2020^45^ | Urothelial Cancer | III | I | OS | ICI + ICI vs CT | Durvalumab + Tremelimumab vs Gemcitabine + Cisplatin/Carboplatin | 342 \ 344 | None |
| KEYNOTE-119 | NCT02555657 | Winer EP et al. 2021^46^ | Breast Cancer | III | >I | OS | ICI vs CT | Pembrolizumab vs Investigator's Choice of Chemotherapy | 312 \ 310 | None |
| ATTRACTION-3 | NCT02569242 | Kato K et al. 2019^47^ | Esophageal Cancer | III | >I | OS | ICI vs CT | Nivolumab vs Investigator's Choice of Chemotherapy | 210 \ 209 | OS or PFS only |
| KEYNOTE-189 | NCT02578680 | Rodriguez-Abreu D et al. 2021^48^ | Non-Small Cell Lung Cancer | III | I | OS+PFS | ICI + CT vs CT | Pembrolizumab + Cisplatin/Carboplatin + Pemetrexed vs Placebo + Cisplatin/Carboplatin + Pemetrexed | 410 \ 206 | OS or PFS only |
| CheckMate 078 | NCT02613507 | Lu S et al. 2021^49^ | Non-Small Cell Lung Cancer | III | >I | OS | ICI vs CT | Nivolumab vs Docetaxel | 338 \ 166 | OS or PFS only |
| JAVELIN Gastric 100 | NCT02625610 | Moehler M et al. 2021^50^ | Gastric Cancer | III | I | OS | ICI vs CT | Avelumab vs Oxaliplatin + Fluoropyrimidine | 249 \ 250 | None |
| IMpower132 | NCT02657434 | Nishio M et al. 2021^51^ | Non-Small Cell Lung Cancer | III | I | OS+PFS | ICI + CT vs CT | Atezolizumab + Pemetrexed + Cisplatin/Carboplatin vs Pemetrexed + Cisplatin/Carboplatin | 292 \ 286 | Both OS and PFS |
| JAVELIN Renal 101 | NCT02684006 | Choueiri TK et al. 2020^52^ | Renal Cancer | III | I | OS+PFS | ICI + anti-angiogenesis agent vs anti-angiogenesis agent | Avelumab + Axitinib vs Sunitinib | 270 \ 290 | Both OS and PFS |
| IMpower133 | NCT02763579 | Horn L et al. 2018^53^ and Liu SV et al. 2021^54^ | Small Cell Lung Cancer | III | I | OS+PFS | ICI + CT vs CT | Atezolizumab + Carboplatin + Etoposide vs Carboplatin + Etoposide | 201 \ 202 | Both OS and PFS |
| KEYNOTE-407 | NCT02775435 | Paz-Ares L et al. 2020^55^ | Non-Small Cell Lung Cancer | III | I | OS+PFS | ICI + CT vs CT | Pembrolizumab + Carboplatin-Paclitaxel/Nab-Paclitaxel vs Placebo + Carboplatin-Paclitaxel/Nab-Paclitaxel | 278 \ 281 | Both OS and PFS |
| IMvigor130 | NCT02807636 | Galsky MD et al. 2020^56^ | Urothelial Cancer | III | I | OS+PFS | ICI + CT vs CT | Atezolizumab + Platinum-based Chemotherapy vs Placebo + Platinum-based Chemotherapy | 451 \ 400 | Both OS and PFS |
| CLEAR | NCT02811861 | Motzer R et al. 2021^57^ | Renal Cancer | III | I | PFS | ICI + anti-angiogenesis agent vs anti-angiogenesis agent | Lenvatinib + Pembrolizumab vs Sunitinib | 355 \ 357 | None |
| KEYNOTE-361 | NCT02853305 | Powles T et al. 2021^58^ | Urothelial Cancer | III | I | OS+PFS | ICI + CT vs CT | Pembrolizumab + Investigator's Choice of Chemotherapy vs Investigator's Choice of Chemotherapy | 351 \ 352 | Both OS and PFS |
| KEYNOTE-361 | NCT02853305 | Powles T et al. 2021^58^ | Urothelial Cancer | III | I | OS+PFS | ICI vs CT | Pembrolizumab vs Investigator's Choice of Chemotherapy | 160 \ 158 | None |
| CO.26 Study | NCT02870920 | Chen EX et al. 2020^59^ | Colorectal Cancer | II | >I | OS | ICI + ICI vs BSC | Durvalumab + Tremelimumab vs Best supportive care | 119 \ 61 | Both OS and PFS |
| CheckMate 649 | NCT02872116 | Janjigian YY et al. 2021^60^ | Gastric Cancer | III | I | OS+PFS | ICI + CT vs CT | Nivolumab + Capecitabine + Oxaliplatin / Nivolumab + Leucovorin + Fluorouracil + Oxaliplatin vs Capecitabine + Oxaliplatin / Leucovorin + Fluorouracil + Oxaliplatin | 473 \ 482 | Both OS and PFS |
| CheckMate 743 | NCT02899299 | Baas P et al. 2021^61^ | Mesothelioma | III | I | OS | ICI + ICI vs CT | Nivolumab + Ipililumab vs Pemetrexed + Cisplatin/Carboplatin | 303 \ 302 | OS or PFS only |
| IMspire150 | NCT02908672 | Gutzmer R et al. 2020^62^ | Melanoma | III | I | PFS | ICI + targeted therapy vs targeted therapy | Atezolizumab + Vemurafenib + Cobimetinib vs Vemurafenib + Cobimetinib | 256 \ 258 | None |
| JAVELIN Head and Neck 100 | NCT02952586 | Lee NY et al. 2021^63^ | Head and Neck Cancer | III | I | PFS | ICI + CT-radio vs CT-radio | Avelumab + Chemoradiotherapy vs Chemoradiotherapy | 350 \ 347 | Both OS and PFS |
| CASPIAN | NCT03043872 | Goldman JW et al. 2020^64^ | Small Cell Lung Cancer | III | I | OS | ICI + ICI + CT vs CT | Durvalumab + Tremelimumab + Platinum-Etoposide vs Platinum-Etoposide | 268 \ 269 | None |
| CASPIAN | NCT03043872 | Goldman JW et al. 2020^64^ | Small Cell Lung Cancer | III | I | OS | ICI + CT vs CT | Durvalumab + Platinum-Etoposide vs Platinum-Etoposide | 268 \ 269 | OS or PFS only |
| EMPOWER-Lung 1 | NCT03088540 | Sezer A et al. 2021^65^ | Non-Small Cell Lung Cancer | III | I | OS+PFS | ICI vs CT | Cemiplimab vs Investigator's Choice of Platinum-based Chemotherapy | 283 \ 280 | OS or PFS only |
| TASUKI-52 | NCT03117049 | Sugawara S et al. 2021^66^ | Non-Small Cell Lung Cancer | III | I | PFS | ICI + CT + anti-angiogenesis agent vs anti-angiogenesis agent + CT | Nivolumab + Carboplatin + Paclitaxel + Bevacizumab vs Placebo + Carboplatin + Paclitaxel + Bevacizumab | 275 \ 275 | Both OS and PFS |
| IMpassion131 | NCT03125902 | Miles D et al. 2021^67^ | Breast Cancer | III | I | PFS | ICI + CT vs CT | Atezolizumab + Paclitaxel vs Placebo + Paclitaxel | 431 \ 220 | Both OS and PFS |
| CameL | NCT03134872 | Zhou C et al. 2021^68^ | Non-Small Cell Lung Cancer | III | I | PFS | ICI + CT vs CT | Camrelizumab + Pemetrexed + Carboplatin vs Pemetrexed + Carboplatin | 205 \ 207 | Both OS and PFS |
| CheckMate 9ER | NCT03141177 | Choueiri TK et al. 2021^69^ | Renal Cancer | III | I | PFS | ICI + anti-angiogenesis agent vs anti-angiogenesis agent | Nivolumab + Cabozantinib vs Sunitinib | 323 \ 328 | Both OS and PFS |
| KEYNOTE-590 | NCT03189719 | Sun JM et al. 2021^70^ | Esophageal Cancer | III | I | OS+PFS | ICI + CT vs CT | Pembrolizumab + Fluorouracil + Cisplatin vs Placebo + Fluorouracil + Cisplatin | 373 \ 376 | Both OS and PFS |
| CheckMate 9LA | NCT03215706 | Reck M et al. 2021^71^ | Non-Small Cell Lung Cancer | III | I | OS | ICI + ICI + CT vs CT | Nivolumab + Ipililumab + Investigator's Choice of Chemotherapy vs Investigator's Choice of Chemotherapy | 361 \ 358 | Both OS and PFS |
|  | NCT03581786 | Mai HQ et al. 2021^72^ | Head and Neck Cancer | III | I | PFS | ICI + CT vs CT | Toripalimab + Gemcitabine/Cisplatin vs Placebo + Gemcitabine/Cisplatin | 146 \ 143 | Both OS and PFS |
| ORIENT-11 | NCT03607539 | Yang Y et al. 2020^73^ | Non-Small Cell Lung Cancer | III | I | PFS | ICI + CT vs CT | Sintilimab + Pemetrexed + Cisplatin/Carboplatin vs Pemetrexed + Cisplatin/Carboplatin | 266 \ 131 | Both OS and PFS |
| ORIENT-12 | NCT03629925 | Zhou C et al. 2021^74^ | Non-Small Cell Lung Cancer | III | I | PFS | ICI + CT vs CT | Sintilimab + Gemcitabine + Platinum-Based Chemotherapy vs Gemcitabine + Platinum-Based Chemotherapy | 179 \ 178 | None |
| ESCORT-1st | NCT03691090 | Luo H et al. 2021^75^ | Esophageal Cancer | III | I | OS+PFS | ICI + CT vs CT | Camrelizumab + Paclitaxel + Cisplatin vs Placebo + Paclitaxel + Cisplatin | 298 \ 298 | Both OS and PFS |

^a^ Grambsch-Therneau test p-value greater than 0.05

### **Table S2.** Quality assessment of trials according to the Cochrane Risk of bias tool

| **Trial** | **NCT** | **Random sequence generation (selection bias)** | **Allocation concealment (selection bias)** | **Blinding of study participants and personnel (performance bias)** | **Blinding of outcome assessors**  **(detection bias)** | **Incomplete outcome data**  **(attrition bias)** | **Selective outcome reporting**  **(reporting bias)** | **Other bias** |
| --- | --- | --- | --- | --- | --- | --- | --- | --- |
|  | NCT00324155 | Low | Low | Low | Low | Low | Low | Low |
|  | NCT01450761 | Low | Low | Low | Low | Low | Low | Low |
| CheckMate 017 | NCT01642004 | Low | Low | High | High | Low | Low | Low |
| CheckMate 025 | NCT01668784 | Low | Low | High | High | Low | Low | Low |
| CheckMate 057 | NCT01673867 | Low | Low | High | High | Low | Low | Low |
| KEYNOTE-002 | NCT01704287 | Low | Low | High | High | Low | Low | Low |
| CheckMate 037 | NCT01721746 | Low | Low | High | High | Low | Low | Low |
| CheckMate 066 | NCT01721772 | Low | Low | Low | Low | Low | Low | Low |
| DETERMINE | NCT01843374 | Low | Low | Low | Low | Low | Low | Low |
| POPLAR | NCT01903993 | Low | Low | High | High | Low | Low | Low |
| KEYNOTE-010 | NCT01905657 | Low | Low | High | High | Low | Low | Low |
| OAK | NCT02008227 | Low | Low | High | High | Low | Low | Low |
| KEYNOTE-021 | NCT02039674 | Low | Low | Low | Low | Low | Low | Low |
| CheckMate 026 | NCT02041533 | Low | Low | High | High | Low | Low | Low |
| CheckMate 141 | NCT02105636 | Low | Low | High | High | Low | Low | Low |
| KEYNOTE-024 | NCT02142738 | Low | Low | High | High | Low | Low | Low |
| KEYNOTE-042 | NCT02220894 | Low | Low | High | High | Low | Low | Low |
| CheckMate 214 | NCT02231749 | Low | Low | High | High | Low | Low | Low |
| KEYNOTE-040 | NCT02252042 | Low | Low | High | High | Low | Low | Low |
| KEYNOTE-045 | NCT02256436 | Low | Low | High | High | Low | Low | Low |
| ATTRACTION-2 | NCT02267343 | Low | Low | Low | Low | Low | Low | Low |
| ARCTIC | NCT02352948 | Low | Low | High | High | Low | Low | Low |
| IMpower150 | NCT02366143 | Low | Low | High | High | Low | Low | Low |
| IMpower130 | NCT02367781 | Low | Low | High | High | Low | Low | Low |
| IMpower131 | NCT02367794 | Low | Low | High | High | Low | Low | Low |
| JAVELIN Lung 200 | NCT02395172 | Low | Low | High | High | Low | Low | Low |
| IMpower110 | NCT02409342 | Low | Low | High | High | Low | Low | Low |
| IMpassion130 | NCT02425891 | Low | Low | Low | Low | Low | Low | Low |
| MYSTIC | NCT02453282 | Low | Low | High | High | Low | Low | Low |
| CheckMate 227 | NCT02477826 | Low | Low | High | High | Low | Low | Low |
| CheckMate 331 | NCT02481830 | Low | Low | High | High | Low | Low | Low |
| DANUBE | NCT02516241 | Low | Low | High | High | Low | Low | Low |
| KEYNOTE-119 | NCT02555657 | Low | Low | High | High | Low | Low | Low |
| ATTRACTION-3 | NCT02569242 | Low | Low | High | High | Low | Low | Low |
| KEYNOTE-189 | NCT02578680 | Low | Low | Low | Low | Low | Low | Low |
| CheckMate 078 | NCT02613507 | Low | Low | High | High | Low | Low | Low |
| JAVELIN Gastric 100 | NCT02625610 | Low | Low | High | High | Low | Low | Low |
| IMpower132 | NCT02657434 | Low | Low | High | High | Low | Low | Low |
| JAVELIN Renal 101 | NCT02684006 | Low | Low | High | High | Low | Low | Low |
| IMpower133 | NCT02763579 | Low | Low | Low | Low | Low | Low | Low |
| KEYNOTE-407 | NCT02775435 | Low | Low | Low | Low | Low | Low | Low |
| IMvigor130 | NCT02807636 | Low | Low | Low | Low | Low | Low | Low |
| CLEAR | NCT02811861 | Low | Low | High | High | Low | Low | Low |
| KEYNOTE-361 | NCT02853305 | Low | Low | High | High | Low | Low | Low |
| CO.26 Study | NCT02870920 | Low | Low | High | High | Low | Low | Low |
| CheckMate 649 | NCT02872116 | Low | Low | High | High | Low | Low | Low |
| CheckMate 743 | NCT02899299 | Low | Low | High | High | Low | Low | Low |
| IMspire150 | NCT02908672 | Low | Low | Low | Low | Low | Low | Low |
| JAVELIN Head and Neck 100 | NCT02952586 | Low | Low | Low | Low | Low | Low | Low |
| CASPIAN | NCT03043872 | Low | Low | High | High | Low | Low | Low |
| EMPOWER-Lung 1 | NCT03088540 | Low | Low | High | High | Low | Low | Low |
| TASUKI-52 | NCT03117049 | Low | Low | Low | Low | Low | Low | Low |
| IMpassion131 | NCT03125902 | Low | Low | Low | Low | Low | Low | Low |
| CameL | NCT03134872 | Low | Low | Low | Low | Low | Low | Low |
| CheckMate 9ER | NCT03141177 | Low | Low | High | High | Low | Low | Low |
| KEYNOTE-590 | NCT03189719 | Low | Low | Low | Low | Low | Low | Low |
| CheckMate 9LA | NCT03215706 | Low | Low | High | High | Low | Low | Low |
|  | NCT03581786 | Low | Low | Low | Low | Low | Low | Low |
| ORIENT-11 | NCT03607539 | Low | Low | Low | Low | Low | Low | Low |
| ORIENT-12 | NCT03629925 | Low | Low | Low | Low | Low | Low | Low |
| ESCORT-1st | NCT03691090 | Low | Low | Low | Low | Low | Low | Low |

### **Table S3.** Treatment effect estimates and their ratios

| **Trial** | **NCT** | **Type of treatment administered in the experimental arm** | **Treatment type (experimental vs control)** | **HR_OS_** | **HR_PFS_** | **HR_PFS_/HR_OS_** | **HR_mPFS_** | **HR_mPFS_/HR_OS_** | **rRMST_PFS_** | **rRMST_PFS_/HR_OS_** | **rRMST_mPFS_** | **rRMST_mPFS_/HR_OS_** |
| --- | --- | --- | --- | --- | --- | --- | --- | --- | --- | --- | --- | --- |
| CheckMate 017 | NCT01642004 | ICI alone | ICI vs CT | 0.61 | 0.63 | 1.04 | 0.53 | 0.87 | 0.65 | 1.07 | 0.56 | 0.91 |
| CheckMate 025 | NCT01668784 | ICI alone | ICI vs targeted therapy | 0.76 | 0.86 | 1.13 | 0.70 | 0.92 | 0.90 | 1.18 | 0.82 | 1.08 |
| CheckMate 057 | NCT01673867 | ICI alone | ICI vs CT | 0.72 | 0.95 | 1.32 | 0.64 | 0.89 | 0.73 | 1.01 | 0.65 | 0.91 |
| KEYNOTE-002 | NCT01704287 | ICI alone | ICI vs CT | 0.86 | 0.60 | 0.69 | 0.88 | 1.02 | 0.73 | 0.85 | 0.92 | 1.07 |
| KEYNOTE-002 | NCT01704287 | ICI alone | ICI vs CT | 0.77 | 0.52 | 0.68 | 0.78 | 1.01 | 0.63 | 0.82 | 0.85 | 1.11 |
| CheckMate 037 | NCT01721746 | ICI alone | ICI vs CT | 0.92 | 0.97 | 1.05 | 0.64 | 0.69 | 0.80 | 0.87 | 0.76 | 0.83 |
| CheckMate 066 | NCT01721772 | ICI alone | ICI vs CT | 0.43 | 0.45 | 1.05 | 0.44 | 1.03 | 0.60 | 1.39 | 0.78 | 1.82 |
| DETERMINE | NCT01843374 | ICI alone | ICI vs placebo | 0.93 | 0.79 | 0.85 | 1.14 | 1.23 | 0.89 | 0.96 | 1.08 | 1.17 |
| POPLAR | NCT01903993 | ICI alone | ICI vs CT | 0.75 | 0.95 | 1.26 | 0.73 | 0.98 | 0.97 | 1.29 | 0.77 | 1.02 |
| KEYNOTE-010 | NCT01905657 | ICI alone | ICI vs CT | 0.67 | 0.79 | 1.18 | 0.67 | 1.00 | 0.59 | 0.88 | 0.58 | 0.87 |
| OAK | NCT02008227 | ICI alone | ICI vs CT | 0.77 | 0.98 | 1.28 | 0.72 | 0.94 | 0.93 | 1.21 | 0.72 | 0.94 |
| CheckMate 026 | NCT02041533 | ICI alone | ICI vs CT | 1.03 | 1.17 | 1.14 | 0.80 | 0.78 | 1.12 | 1.08 | 0.88 | 0.85 |
| CheckMate 141 | NCT02105636 | ICI alone | ICI vs CT | 0.68 | 0.89 | 1.31 | 0.65 | 0.96 | 0.90 | 1.32 | 0.78 | 1.15 |
| KEYNOTE-024 | NCT02142738 | ICI alone | ICI vs CT | 0.62 | 0.49 | 0.78 | 0.51 | 0.82 | 0.69 | 1.11 | 0.69 | 1.11 |
| KEYNOTE-042 | NCT02220894 | ICI alone | ICI vs CT | 0.80 | 1.05 | 1.31 | 0.85 | 1.06 | 1.03 | 1.28 | 0.89 | 1.11 |
| KEYNOTE-040 | NCT02252042 | ICI alone | ICI vs CT | 0.78 | 0.91 | 1.17 | 0.77 | 0.99 | 0.92 | 1.18 | 0.84 | 1.08 |
| KEYNOTE-045 | NCT02256436 | ICI alone | ICI vs CT | 0.73 | 0.94 | 1.30 | 0.63 | 0.87 | 0.91 | 1.25 | 0.79 | 1.09 |
| ATTRACTION-2 | NCT02267343 | ICI alone | ICI vs placebo | 0.65 | 0.62 | 0.96 | 0.66 | 1.02 | 0.59 | 0.91 | 0.75 | 1.16 |
| ARCTIC | NCT02352948 | ICI alone | ICI vs CT | 0.63 | 0.71 | 1.12 | 0.67 | 1.05 | 0.71 | 1.11 | 0.76 | 1.19 |
| JAVELIN Lung 200 | NCT02395172 | ICI alone | ICI vs CT | 0.87 | 1.02 | 1.17 | 0.63 | 0.72 | 0.99 | 1.13 | 0.82 | 0.94 |
| IMpower110 | NCT02409342 | ICI alone | ICI vs CT | 0.60 | 0.58 | 0.98 | 0.58 | 0.98 | 0.71 | 1.19 | 0.77 | 1.29 |
| MYSTIC | NCT02453282 | ICI alone | ICI vs CT | 0.75 | 0.90 | 1.19 | 0.61 | 0.80 | 0.98 | 1.29 | 0.75 | 1.00 |
| CheckMate 331 | NCT02481830 | ICI alone | ICI vs CT | 0.87 | 1.47 | 1.68 | 0.72 | 0.83 | 1.23 | 1.42 | 0.76 | 0.87 |
| DANUBE | NCT02516241 | ICI alone | ICI vs CT | 0.89 | 1.24 | 1.40 | 0.74 | 0.83 | 0.97 | 1.09 | 0.73 | 0.82 |
| KEYNOTE-119 | NCT02555657 | ICI alone | ICI vs CT | 0.98 | 1.83 | 1.86 | 0.75 | 0.77 | 1.49 | 1.52 | 0.78 | 0.79 |
| ATTRACTION-3 | NCT02569242 | ICI alone | ICI vs CT | 0.76 | 1.08 | 1.41 | 0.60 | 0.78 | 0.98 | 1.28 | 0.70 | 0.92 |
| CheckMate 078 | NCT02613507 | ICI alone | ICI vs CT | 0.75 | 0.80 | 1.06 | 0.63 | 0.84 | 0.72 | 0.96 | 0.70 | 0.94 |
| JAVELIN Gastric 100 | NCT02625610 | ICI alone | ICI vs CT | 0.91 | 1.06 | 1.17 | 0.88 | 0.98 | 0.94 | 1.04 | 0.91 | 1.00 |
| KEYNOTE-361 | NCT02853305 | ICI alone | ICI vs CT | 1.01 | 1.31 | 1.29 | 0.78 | 0.77 | 1.12 | 1.11 | 0.83 | 0.82 |
| EMPOWER-Lung 1 | NCT03088540 | ICI alone | ICI vs CT | 0.57 | 0.56 | 0.98 | 0.43 | 0.74 | 0.69 | 1.20 | 0.64 | 1.12 |
|  | NCT00324155 | ICI + CT | ICI + CT vs CT | 0.76 | 0.81 | 1.06 | 0.79 | 1.03 | 0.75 | 0.99 | 0.82 | 1.08 |
|  | NCT01450761 | ICI + CT | ICI + CT vs CT | 0.96 | 0.88 | 0.92 | 0.96 | 1.00 | 0.93 | 0.97 | 0.98 | 1.02 |
| KEYNOTE-021 | NCT02039674 | ICI + CT | ICI + CT vs CT | 0.76 | 0.56 | 0.74 | 0.62 | 0.81 | 0.69 | 0.91 | 0.76 | 1.00 |
| IMpower130 | NCT02367781 | ICI + CT | ICI + CT vs CT | 0.76 | 0.63 | 0.83 | 0.72 | 0.94 | 0.70 | 0.92 | 0.80 | 1.06 |
| IMpower131 | NCT02367794 | ICI + CT | ICI + CT vs CT | 0.86 | 0.69 | 0.81 | 0.81 | 0.94 | 0.78 | 0.90 | 0.85 | 0.99 |
| IMpassion130 | NCT02425891 | ICI + CT | ICI + CT vs CT | 0.69 | 0.66 | 0.96 | 0.74 | 1.07 | 0.70 | 1.02 | 0.81 | 1.18 |
| KEYNOTE-189 | NCT02578680 | ICI + CT | ICI + CT vs CT | 0.59 | 0.49 | 0.83 | 0.52 | 0.89 | 0.56 | 0.95 | 0.63 | 1.07 |
| IMpower132 | NCT02657434 | ICI + CT | ICI + CT vs CT | 0.86 | 0.57 | 0.66 | 0.66 | 0.76 | 0.62 | 0.72 | 0.73 | 0.85 |
| IMpower133 | NCT02763579 | ICI + CT | ICI + CT vs CT | 0.77 | 0.74 | 0.97 | 0.73 | 0.95 | 0.85 | 1.10 | 0.82 | 1.07 |
| KEYNOTE-407 | NCT02775435 | ICI + CT | ICI + CT vs CT | 0.73 | 0.59 | 0.80 | 0.63 | 0.86 | 0.67 | 0.92 | 0.73 | 1.00 |
| IMvigor130 | NCT02807636 | ICI + CT | ICI + CT vs CT | 0.84 | 0.81 | 0.96 | 0.80 | 0.95 | 0.88 | 1.05 | 0.88 | 1.05 |
| KEYNOTE-361 | NCT02853305 | ICI + CT | ICI + CT vs CT | 0.87 | 0.77 | 0.89 | 0.71 | 0.82 | 0.83 | 0.96 | 0.81 | 0.93 |
| CheckMate 649 | NCT02872116 | ICI + CT | ICI + CT vs CT | 0.70 | 0.70 | 0.99 | 0.72 | 1.03 | 0.76 | 1.09 | 0.80 | 1.13 |
| JAVELIN Head and Neck 100 | NCT02952586 | ICI + CT | ICI + CT-radio vs CT-radio | 1.26 | 1.18 | 0.94 | 1.18 | 0.94 | 1.05 | 0.83 | 1.04 | 0.83 |
| CASPIAN | NCT03043872 | ICI + CT | ICI + CT vs CT | 0.76 | 0.78 | 1.03 | 0.85 | 1.13 | 0.80 | 1.05 | 0.86 | 1.14 |
| IMpassion131 | NCT03125902 | ICI + CT | ICI + CT vs CT | 1.16 | 0.88 | 0.76 | 0.95 | 0.82 | 0.93 | 0.80 | 0.97 | 0.83 |
| CameL | NCT03134872 | ICI + CT | ICI + CT vs CT | 0.72 | 0.60 | 0.83 | 0.66 | 0.91 | 0.80 | 1.11 | 0.87 | 1.20 |
| KEYNOTE-590 | NCT03189719 | ICI + CT | ICI + CT vs CT | 0.72 | 0.65 | 0.90 | 0.66 | 0.91 | 0.74 | 1.03 | 0.75 | 1.04 |
|  | NCT03581786 | ICI + CT | ICI + CT vs CT | 0.63 | 0.52 | 0.83 | 0.53 | 0.85 | 0.81 | 1.30 | 0.83 | 1.32 |
| ORIENT-11 | NCT03607539 | ICI + CT | ICI + CT vs CT | 0.60 | 0.47 | 0.79 | 0.52 | 0.87 | 0.74 | 1.23 | 0.82 | 1.37 |
| ORIENT-12 | NCT03629925 | ICI + CT | ICI + CT vs CT | 0.56 | 0.54 | 0.96 | 0.50 | 0.88 | 0.73 | 1.29 | 0.74 | 1.31 |
| ESCORT-1st | NCT03691090 | ICI + CT | ICI + CT vs CT | 0.71 | 0.57 | 0.80 | 0.65 | 0.91 | 0.75 | 1.05 | 0.82 | 1.15 |
| CheckMate 214 | NCT02231749 | ICI + ICI or other treatment(s) | ICI + ICI vs anti-angiogenesis agent | 0.66 | 0.82 | 1.24 | 0.72 | 1.08 | 0.89 | 1.34 | 0.85 | 1.29 |
| ARCTIC | NCT02352948 | ICI + ICI or other treatment(s) | ICI + ICI vs CT | 0.78 | 0.76 | 0.97 | 0.65 | 0.83 | 0.74 | 0.95 | 0.75 | 0.96 |
| IMpower150 | NCT02366143 | ICI + ICI or other treatment(s) | ICI + CT + anti-angiogenesis agent vs anti-angiogenesis agent + CT | 0.80 | 0.60 | 0.75 | 0.69 | 0.86 | 0.75 | 0.93 | 0.79 | 0.98 |
| MYSTIC | NCT02453282 | ICI + ICI or other treatment(s) | ICI + ICI vs CT | 0.86 | 1.05 | 1.23 | 0.74 | 0.86 | 1.07 | 1.25 | 0.83 | 0.97 |
| CheckMate 227 | NCT02477826 | ICI + ICI or other treatment(s) | ICI + ICI vs CT | 0.78 | 0.83 | 1.06 | 0.60 | 0.76 | 0.73 | 0.93 | 0.65 | 0.83 |
| DANUBE | NCT02516241 | ICI + ICI or other treatment(s) | ICI + ICI vs CT | 0.85 | 1.06 | 1.25 | 0.70 | 0.82 | 0.88 | 1.04 | 0.69 | 0.82 |
| JAVELIN Renal 101 | NCT02684006 | ICI + ICI or other treatment(s) | ICI + anti-angiogenesis agent vs anti-angiogenesis agent | 0.85 | 0.64 | 0.76 | 0.90 | 1.07 | 0.79 | 0.94 | 0.96 | 1.14 |
| CLEAR | NCT02811861 | ICI + ICI or other treatment(s) | ICI + anti-angiogenesis agent vs anti-angiogenesis agent | 0.71 | 0.42 | 0.59 | 0.46 | 0.65 | 0.64 | 0.90 | 0.71 | 1.00 |
| CO.26 Study | NCT02870920 | ICI + ICI or other treatment(s) | ICI + ICI vs BSC | 0.69 | 0.93 | 1.35 | 0.73 | 1.05 | 0.94 | 1.35 | 0.85 | 1.23 |
| CheckMate 743 | NCT02899299 | ICI + ICI or other treatment(s) | ICI + ICI vs CT | 0.75 | 0.98 | 1.30 | 0.70 | 0.93 | 0.92 | 1.23 | 0.76 | 1.02 |
| IMspire150 | NCT02908672 | ICI + ICI or other treatment(s) | ICI + targeted therapy vs targeted therapy | 0.83 | 0.76 | 0.91 | 0.75 | 0.90 | 0.87 | 1.04 | 0.87 | 1.05 |
| CASPIAN | NCT03043872 | ICI + ICI or other treatment(s) | ICI + ICI + CT vs CT | 0.83 | 0.83 | 1.01 | 0.85 | 1.03 | 0.83 | 1.01 | 0.85 | 1.03 |
| TASUKI-52 | NCT03117049 | ICI + ICI or other treatment(s) | ICI + CT + anti-angiogenesis agent vs anti-angiogenesis agent + CT | 0.86 | 0.58 | 0.67 | 0.63 | 0.73 | 0.75 | 0.87 | 0.79 | 0.93 |
| CheckMate 9ER | NCT03141177 | ICI + ICI or other treatment(s) | ICI + anti-angiogenesis agent vs anti-angiogenesis agent | 0.59 | 0.52 | 0.88 | 0.57 | 0.96 | 0.72 | 1.22 | 0.78 | 1.32 |
| CheckMate 9LA | NCT03215706 | ICI + ICI or other treatment(s) | ICI + ICI + CT vs CT | 0.73 | 0.69 | 0.95 | 0.61 | 0.84 | 0.71 | 0.97 | 0.69 | 0.95 |

### **Table S4.** Pairwise agreement between statistical significance of HR_OS_ and each surrogate measure

|  | **HR_OS_** | **HR_PFS_** | **HR_mPFS_** | **rRMST_PFS_** | **rRMST_mPFS_** |
| --- | --- | --- | --- | --- | --- |
| **ICI alone** *(n=30 comparisons)* |  |  |  |  |  |
| N of significant results (%) ^a^ | 19 (63) | 14 (47) | 25 (83) | 12 (40) | 25 (83) |
| Cohen’s Kappa coefficient (95% CI) | - | 0.02 (-0.32, 0.36) | 0.51 (0.21, 0.82) | 0.30 (0.01, 0.60) | 0.35 (0.03, 0.68) |
| McNemar’s p-value | - | 0.197 | 0.014 | 0.035 | 0.034 |
| **ICI + CT** *(n=22 comparisons)* |  |  |  |  |  |
| N of significant results ^a^ | 13 (59) | 19 (86) | 18 (82) | 19 (86) | 19 (86) |
| Cohen’s Kappa coefficient (95% CI) | - | 0.37 (0.04, 0.71) | 0.28 (-0.09, 0.65) | 0.37 (0.04, 0.71) | 0.37 (0.04, 0.71) |
| McNemar’s p-value | - | 0.014 | 0.059 | 0.014 | 0.014 |
| **ICI + ICI or other treatment(s)** *(n=15 comparisons)* |  |  |  |  |  |
| N of significant results ^a^ | 9 (60) | 11 (73) | 12 (80) | 11 (73) | 13 (87) |
| Cohen’s Kappa coefficient (95% CI) | - | 0.12 (-0.37, 0.61) | -0.06 (-0.50, 0.38) | 0.12 (-0.37, 0.61) | 0.06 (-0.34, 0.47) |
| McNemar’s p-value | - | 0.414 | 0.257 | 0.414 | 0.103 |

^a^ P-value < 0.05 was considered statistically significant

## **REFERENCES**

**1**. Grambsch PM, Therneau TM: Proportional hazards tests and diagnostics based on weighted residuals. Biometrika 81:515–526, 1994

**2**. Zhao L, Claggett B, Tian L, et al: On the Restricted Mean Survival Time Curve in Survival Analysis. Biometrics 72:215–221, 2016

**3**. Higgins JPT, Thompson SG: Quantifying heterogeneity in a meta-analysis. Stat Med 21:1539–1558, 2002

**4**. Buyse M, Molenberghs G, Burzykowski T, et al: The validation of surrogate endpoints in meta-analyses of randomized experiments. Biostatistics 1:49–67, 2000

**5**. Xie W, Halabi S, Tierney JF, et al: A Systematic Review and Recommendation for Reporting of Surrogate Endpoint Evaluation Using Meta-analyses. JNCI Cancer Spectr 3:pkz002, 2019

**6**. Burzykowski T, Buyse M: Surrogate threshold effect: an alternative measure for meta-analytic surrogate endpoint validation. Pharm Stat 5:173–186, 2006

**7**. Greenland S: Quantitative methods in the review of epidemiologic literature. Epidemiol Rev 9:1–30, 1987

**8**. Spigel DR, Vicente D, Ciuleanu TE, et al: Second-line nivolumab in relapsed small-cell lung cancer: CheckMate 331. Ann Oncol 32:631–641, 2021

**9**. Alexander BM, Schoenfeld JD, Trippa L: Hazards of Hazard Ratios - Deviations from Model Assumptions in Immunotherapy. N Engl J Med 378:1158–1159, 2018

**10**. Robert C, Thomas L, Bondarenko I, et al: Ipilimumab plus Dacarbazine for Previously Untreated Metastatic Melanoma. N Engl J Med 364:2517–2526, 2011

**11**. Reck M, Luft A, Szczesna A, et al: Phase III randomized trial of ipilimumab plus etoposide and platinum versus placebo plus etoposide and platinum in extensive-stage small-cell lung cancer. J Clin Oncol 34:3740–3748, 2016

**12**. Borghaei H, Gettinger S, Vokes EE, et al: Five-Year Outcomes From the Randomized , Phase III Trials CheckMate 017 and 057: Nivolumab Versus Docetaxel in Previously Treated Non–Small-Cell Lung Cancer. J Clin Oncol 39:723–733, 2021

**13**. Motzer RJ, Escudier B, McDermott DF, et al: Nivolumab versus Everolimus in Advanced Renal-Cell Carcinoma. N Engl J Med 373:1803–1813, 2015

**14**. Ribas A, Puzanov I, Dummer R, et al: Pembrolizumab versus investigator-choice chemotherapy for ipilimumab-refractory melanoma (KEYNOTE-002): a randomised, controlled, phase 2 trial. Lancet Oncol 16:908–918, 2015

**15**. Hamid O, Puzanov I, Dummer R, et al: Final analysis of a randomised trial comparing pembrolizumab versus investigator-choice chemotherapy for ipilimumab-refractory advanced melanoma. Eur J Cancer 86:37–45, 2017

**16**. Larkin J, Minor D, D’Angelo S, et al: Overall Survival in Patients With Advanced Melanoma Who Received Nivolumab Versus Investigator’s Choice Chemotherapy in CheckMate 037: A Randomized, Controlled, Open-Label Phase III Trial. J Clin Oncol 36:383–390, 2018

**17**. Robert C, Long G V, Brady B, et al: Nivolumab in Previously Untreated Melanoma without BRAF Mutation. N Engl J Med 372:320–330, 2015

**18**. Maio M, Scherpereel A, Calabrò L, et al: Tremelimumab as second-line or third-line treatment in relapsed malignant mesothelioma (DETERMINE): a multicentre, international, randomised, double-blind, placebo-controlled phase 2b trial. Lancet Oncol 18:1261–1273, 2017

**19**. Fehrenbacher L, Spira A, Ballinger M, et al: Atezolizumab versus docetaxel for patients with previously treated non-small-cell lung cancer (POPLAR): a multicentre, open-label, phase 2 randomised controlled trial. Lancet 387:1837–1846, 2016

**20**. Mazieres J, Rittmeyer A, Gadgeel S, et al: Atezolizumab Versus Docetaxel in Pretreated Patients With NSCLC: Final Results From the Randomized Phase 2 POPLAR and Phase 3 OAK Clinical Trials. J Thorac Oncol 16:140–150, 2020

**21**. Herbst RS, Garon EB, Kim DW, et al: Five Year Survival Update From KEYNOTE-010: Pembrolizumab Versus Docetaxel for Previously Treated, Programmed Death-Ligand 1-Positive Advanced NSCLC. J Thorac Oncol 16:1718–1732, 2021

**22**. Fehrenbacher L, von Pawel J, Park K, et al: Updated Efficacy Analysis Including Secondary Population Results for OAK: A Randomized Phase III Study of Atezolizumab versus Docetaxel in Patients with Previously Treated Advanced Non-Small Cell Lung Cancer. J Thorac Oncol 13:1156–1170, 2018

**23**. Awad MM, Gadgeel SM, Borghaei H, et al: Long-Term Overall Survival From KEYNOTE-021 Cohort G: Pemetrexed and Carboplatin With or Without Pembrolizumab as First-Line Therapy for Advanced Nonsquamous NSCLC. J Thorac Oncol 16:162–168, 2021

**24**. Carbone DP, Reck M, Paz-Ares L, et al: First-Line Nivolumab in Stage IV or Recurrent Non–Small-Cell Lung Cancer. N Engl J Med 376:2415–2426, 2017

**25**. Ferris RL, Blumenschein G, Fayette J, et al: Nivolumab for Recurrent Squamous-Cell Carcinoma of the Head and Neck. N Engl J Med 375:1856–1867, 2016

**26**. Reck M, Rodriguez-Abreu D, Robinson AG, et al: Pembrolizumab versus Chemotherapy for PD-L1-Positive Non-Small-Cell Lung Cancer. N Engl J Med 375:1823–1833, 2016

**27**. Reck M, Rodríguez-Abreu D, Robinson AG, et al: Updated analysis of KEYNOTE-024: Pembrolizumab versus platinum-based chemotherapy for advanced non–small-cell lung cancer with PD-L1 tumor proportion score of 50% or greater. J Clin Oncol 37:537–546, 2019

**28**. Mok TSK, Wu YL, Kudaba I, et al: Pembrolizumab versus chemotherapy for previously untreated, PD-L1-expressing, locally advanced or metastatic non-small-cell lung cancer (KEYNOTE-042): a randomised, open-label, controlled, phase 3 trial. Lancet 393:1819–1830, 2019

**29**. Motzer RJ, Tannir NM, McDermott DF, et al: Nivolumab plus Ipilimumab versus Sunitinib in Advanced Renal-Cell Carcinoma. N Engl J Med 378:1277–1290, 2018

**30**. Cohen EEW, Soulières D, Le Tourneau C, et al: Pembrolizumab versus methotrexate, docetaxel, or cetuximab for recurrent or metastatic head-and-neck squamous cell carcinoma (KEYNOTE-040): a randomised, open-label, phase 3 study. Lancet 393:156–167, 2019

**31**. Bellmunt J, de Wit R, Vaughn DJ, et al: Pembrolizumab as Second-Line Therapy for Advanced Urothelial Carcinoma. N Engl J Med 376:1015–1026, 2017

**32**. Chen LT, Satoh T, Ryu MH, et al: A phase 3 study of nivolumab in previously treated advanced gastric or gastroesophageal junction cancer (ATTRACTION-2): 2-year update data. Gastric Cancer 23:510–519, 2020

**33**. Planchard D, Reinmuth N, Orlov S, et al: ARCTIC: durvalumab with or without tremelimumab as third-line or later treatment of metastatic non-small-cell lung cancer. Ann Oncol 31:609–618, 2020

**34**. Socinski MA, Jotte RM, Cappuzzo F, et al: Atezolizumab for First-Line Treatment of Metastatic Nonsquamous NSCLC. N Engl J Med 378:2288–2301, 2018

**35**. Socinski MA, Nishio M, Jotte RM, et al: IMpower150 Final Overall Survival Analyses for Atezolizumab Plus Bevacizumab and Chemotherapy in First-Line Metastatic Nonsquamous NSCLC. J Thorac Oncol 16:1909–1924, 2021

**36**. West H, McCleod M, Hussein M, et al: Atezolizumab in combination with carboplatin plus nab-paclitaxel chemotherapy compared with chemotherapy alone as first-line treatment for metastatic non-squamous non-small-cell lung cancer (IMpower130): a multicentre, randomised, open-label, phase 3 tria. Lancet Oncol 20:924–937, 2019

**37**. Jotte R, Cappuzzo F, Vynnychenko I, et al: Atezolizumab in Combination With Carboplatin and Nab-Paclitaxel in Advanced Squamous NSCLC (IMpower131): Results From a Randomized Phase III Trial. J Thorac Oncol 15:1351–1360, 2020

**38**. Barlesi F, Vansteenkiste J, Spigel D, et al: Avelumab versus docetaxel in patients with platinum-treated advanced non-small-cell lung cancer (JAVELIN Lung 200): an open-label, randomised, phase 3 study. Lancet Oncol 19:1468–1479, 2018

**39**. Park K, Özgüroğlu M, Vansteenkiste J, et al: Avelumab Versus Docetaxel in Patients With Platinum-Treated Advanced NSCLC: 2-Year Follow-Up From the JAVELIN Lung 200 Phase 3 Trial. J Thorac Oncol 16:1369–1378, 2021

**40**. Herbst RS, Giaccone G, de Marinis F, et al: Atezolizumab for First-Line Treatment of PD-L1–Selected Patients with NSCLC. N Engl J Med 383:1328–1339, 2020

**41**. Emens LA, Adams S, Barrios CH, et al: First-line atezolizumab plus nab-paclitaxel for unresectable, locally advanced, or metastatic triple-negative breast cancer: IMpassion130 final overall survival analysis. Ann Oncol 32:983–993, 2021

**42**. Rizvi NA, Cho BC, Reinmuth N, et al: Durvalumab with or Without Tremelimumab vs Standard Chemotherapy in First-line Treatment of Metastatic Non-Small Cell Lung Cancer: The MYSTIC Phase 3 Randomized Clinical Trial. JAMA Oncol 6:661–674, 2020

**43**. Hellmann MD, Paz-Ares L, Bernabe Caro R, et al: Nivolumab plus Ipilimumab in Advanced Non–Small-Cell Lung Cancer. N Engl J Med 381:2020–2031, 2019

**44**. Paz-Ares LG, Ciuleanu TE, Lee JS, et al: Nivolumab (NIVO) plus ipilimumab (IPI) versus chemotherapy (chemo) as first-line (1L) treatment for advanced non-small cell lung cancer (NSCLC): 4-year update from CheckMate 227. J Clin Oncol 39:9016, 2021

**45**. Powles T, van der Heijden MS, Castellano D, et al: Durvalumab alone and durvalumab plus tremelimumab versus chemotherapy in previously untreated patients with unresectable, locally advanced or metastatic urothelial carcinoma (DANUBE): a randomised, open-label, multicentre, phase 3 trial. Lancet Oncol 21:1574–1588, 2020

**46**. Winer EP, Lipatov O, Im SA, et al: Pembrolizumab versus investigator-choice chemotherapy for metastatic triple-negative breast cancer (KEYNOTE-119): a randomised, open-label, phase 3 trial. Lancet Oncol 22:499–511, 2021

**47**. Kato K, Cho BC, Takahashi M, et al: Nivolumab versus chemotherapy in patients with advanced oesophageal squamous cell carcinoma refractory or intolerant to previous chemotherapy (ATTRACTION-3): a multicentre, randomised, open-label, phase 3 trial. Lancet Oncol 20:1506–1517, 2019

**48**. Rodríguez-Abreu D, Powell SF, Hochmair MJ, et al: Pemetrexed plus platinum with or without pembrolizumab in patients with previously untreated metastatic nonsquamous NSCLC: protocol-specified final analysis from KEYNOTE-189. Ann Oncol 32:881–895, 2021

**49**. Lu S, Wang J, Cheng Y, et al: Nivolumab versus docetaxel in a predominantly Chinese patient population with previously treated advanced non-small cell lung cancer: 2-year follow-up from a randomized, open-label, phase 3 study (CheckMate 078). Lung Cancer 152:7–14, 2021

**50**. Moehler M, Dvorkin M, Boku N, et al: Phase III Trial of Avelumab Maintenance After First-Line Induction Chemotherapy Versus Continuation of Chemotherapy in Patients With Gastric Cancers: Results From JAVELIN Gastric 100. J Clin Oncol 39:966–977, 2021

**51**. Nishio M, Barlesi F, West H, et al: Atezolizumab Plus Chemotherapy for First-Line Treatment of Nonsquamous NSCLC: Results From the Randomized Phase 3 IMpower132 Trial. J Thorac Oncol 16:653–664, 2021

**52**. Choueiri TK, Motzer RJ, Rini BI, et al: Updated efficacy results from the JAVELIN Renal 101 trial: first-line avelumab plus axitinib versus sunitinib in patients with advanced renal cell carcinoma. Ann Oncol 31:1030–1039, 2020

**53**. Horn L, Mansfield AS, Szczęsna A, et al: First-Line Atezolizumab plus Chemotherapy in Extensive-Stage Small-Cell Lung Cancer. N Engl J Med 379:2220–2229, 2018

**54**. Liu S V, Reck M, Mansfield AS, et al: Updated Overall Survival and PD-L1 Subgroup Analysis of Patients With Extensive-Stage Small-Cell Lung Cancer Treated With Atezolizumab, Carboplatin, and Etoposide (IMpower133). J Clin Oncol 39:619–630, 2021

**55**. Paz-Ares L, Vicente D, Tafreshi A, et al: A Randomized, Placebo-Controlled Trial of Pembrolizumab Plus Chemotherapy in Patients With Metastatic Squamous NSCLC: Protocol-Specified Final Analysis of KEYNOTE-407. J Thorac Oncol 15:1657–1669, 2020

**56**. Galsky MD, Arija JÁA, Bamias A, et al: Atezolizumab with or without chemotherapy in metastatic urothelial cancer (IMvigor130): a multicentre, randomised, placebo-controlled phase 3 trial. Lancet 395:1547–1557, 2020

**57**. Motzer R, Alekseev B, Rha SY, et al: Lenvatinib plus Pembrolizumab or Everolimus for Advanced Renal Cell Carcinoma. N Engl J Med 384:1289–1300, 2021

**58**. Powles T, Csőszi T, Özgüroğlu M, et al: Pembrolizumab alone or combined with chemotherapy versus chemotherapy as first-line therapy for advanced urothelial carcinoma (KEYNOTE-361): a randomised, open-label, phase 3 trial. Lancet Oncol 22:931–945, 2021

**59**. Chen EX, Jonker DJ, Loree JM, et al: Effect of Combined Immune Checkpoint Inhibition vs Best Supportive Care Alone in Patients with Advanced Colorectal Cancer: The Canadian Cancer Trials Group CO.26 Study. JAMA Oncol 6:831–838, 2020

**60**. Janjigian YY, Shitara K, Moehler M, et al: First-line nivolumab plus chemotherapy versus chemotherapy alone for advanced gastric, gastro-oesophageal junction, and oesophageal adenocarcinoma (CheckMate 649): a randomised, open-label, phase 3 trial. Lancet 398:27–40, 2021

**61**. Baas P, Scherpereel A, Nowak AK, et al: First-line nivolumab plus ipilimumab in unresectable malignant pleural mesothelioma (CheckMate 743): a multicentre, randomised, open-label, phase 3 trial. Lancet 397:375–386, 2021

**62**. Gutzmer R, Stroyakovskiy D, Gogas H, et al: Atezolizumab, vemurafenib, and cobimetinib as first-line treatment for unresectable advanced BRAFV600 mutation-positive melanoma (IMspire150): primary analysis of the randomised, double-blind, placebo-controlled, phase 3 trial. Lancet 395:1835–1844, 2020

**63**. Lee NY, Ferris RL, Psyrri A, et al: Avelumab plus standard-of-care chemoradiotherapy versus chemoradiotherapy alone in patients with locally advanced squamous cell carcinoma of the head and neck: a randomised, double-blind, placebo-controlled, multicentre, phase 3 trial. Lancet Oncol 22:450–462, 2021

**64**. Goldman JW, Dvorkin M, Chen Y, et al: Durvalumab, with or without tremelimumab, plus platinum–etoposide versus platinum–etoposide alone in first-line treatment of extensive-stage small-cell lung cancer (CASPIAN): updated results from a randomised, controlled, open-label, phase 3 trial. Lancet Oncol 22:51–65, 2021

**65**. Sezer A, Kilickap S, Gümüş M, et al: Cemiplimab monotherapy for first-line treatment of advanced non-small-cell lung cancer with PD-L1 of at least 50%: a multicentre, open-label, global, phase 3, randomised, controlled trial. Lancet 397:592–604, 2021

**66**. Sugawara S, Lee JS, Kang JH, et al: Nivolumab with carboplatin, paclitaxel, and bevacizumab for first-line treatment of advanced nonsquamous non-small-cell lung cancer. Ann Oncol 32:1137–1147, 2021

**67**. Miles D, Gligorov J, André F, et al: Primary results from IMpassion131, a double-blind, placebo-controlled, randomised phase III trial of first-line paclitaxel with or without atezolizumab for unresectable locally advanced/metastatic triple-negative breast cancer. Ann Oncol 32:994–1004, 2021

**68**. Zhou C, Chen G, Huang Y, et al: Camrelizumab plus carboplatin and pemetrexed versus chemotherapy alone in chemotherapy-naive patients with advanced non-squamous non-small-cell lung cancer (CameL): a randomised, open-label, multicentre, phase 3 trial. Lancet Respir Med 9:305–314, 2021

**69**. Choueiri TK, Powles T, Burotto M, et al: Nivolumab plus Cabozantinib versus Sunitinib for Advanced Renal-Cell Carcinoma. N Engl J Med 384:829–841, 2021

**70**. Sun JM, Shen L, Shah MA, et al: Pembrolizumab plus chemotherapy versus chemotherapy alone for first-line treatment of advanced oesophageal cancer (KEYNOTE-590): a randomised, placebo-controlled, phase 3 study. Lancet 398:759–771, 2021

**71**. Reck M, Ciuleanu TE, Cobo M, et al: First-line nivolumab plus ipilimumab with two cycles of chemotherapy versus chemotherapy alone (four cycles) in advanced non-small-cell lung cancer: CheckMate 9LA 2-year update. ESMO Open 6:100273, 2021

**72**. Mai HQ, Chen QY, Chen D, et al: Toripalimab or placebo plus chemotherapy as first-line treatment in advanced nasopharyngeal carcinoma: a multicenter randomized phase 3 trial. Nat Med 27:1536–1543, 2021

**73**. Yang Y, Wang Z, Fang J, et al: Efficacy and Safety of Sintilimab Plus Pemetrexed and Platinum as First-Line Treatment for Locally Advanced or Metastatic Nonsquamous NSCLC: a Randomized, Double-Blind, Phase 3 Study (Oncology pRogram by InnovENT anti-PD-1-11). J Thorac Oncol 15:1636–1646, 2020

**74**. Zhou C, Wu L, Fan Y, et al: Sintilimab Plus Platinum and Gemcitabine as First-Line Treatment for Advanced or Metastatic Squamous NSCLC: Results From a Randomized, Double-Blind, Phase 3 Trial (ORIENT-12). J Thorac Oncol 16:1501–1511, 2021

**75**. Luo H, Lu J, Bai Y, et al: Effect of Camrelizumab vs Placebo Added to Chemotherapy on Survival and Progression-Free Survival in Patients with Advanced or Metastatic Esophageal Squamous Cell Carcinoma: The ESCORT-1st Randomized Clinical Trial. JAMA 326:916–925, 2021
